# Iron chelation as a new therapeutic approach to prevent senescence and liver fibrosis progression

**DOI:** 10.1038/s41419-024-07063-0

**Published:** 2024-09-17

**Authors:** Josep Amengual, Ania Alay, Javier Vaquero, Ester Gonzalez-Sanchez, Esther Bertran, Aránzazu Sánchez, Blanca Herrera, Kathleen Meyer, Mate Maus, Manuel Serrano, María Luz Martínez-Chantar, Isabel Fabregat

**Affiliations:** 1 TGF-β and Cancer Group, Oncobell Program, Bellvitge Biomedical Research Institute (IDIBELL), L’Hospitalet de Llobregat, Barcelona, Spain; 2 Centro de Investigación Biomédica en Red de Enfermedades Hepáticas y Digestivas (CIBERehd), Madrid, Spain; 3 Unit of Bioinformatics for Precision Oncology, Catalan Institute of Oncology (ICO), L’Hospitalet de Llobregat, Barcelona, Spain; 4 Preclinical and Experimental Research in Thoracic Tumors (PReTT), Oncobell Program, IDIBELL, L’Hospitalet de Llobregat, Barcelona, Spain; 5 HepatoBiliary Tumours Lab, Centro de Investigación del Cáncer and Instituto de Biología Molecular y Celular del Cáncer, CSIC-Universidad de Salamanca, Salamanca, 37007 Spain; 6 Department of Physiology and Pharmacology, University of Salamanca, 37007 Salamanca, Spain; 7 Department of Biochemistry and Molecular Biology, Faculty of Pharmacy, Complutense University of Madrid, Madrid, Spain; 8 Health Research Institute of the “Hospital Clínico San Carlos” (IdISSC), Madrid, Spain; 9 Institute for Research in Biomedicine (IRB Barcelona), The Barcelona Institute of Science and Technology (BIST), Barcelona, Spain; 10 Altos Labs, Cambridge Institute of Science, Cambridge, United Kingdom; 11 Vall d’Hebron Institute of Oncology, Barcelona, Spain; 12 Catalan Institution for Research and Advanced Studies (ICREA), Barcelona, Spain; 13 Liver Disease and Liver Metabolism Laboratory, CIC bioGUNE-BRTA (Basque Research & Technology Alliance), Derio, Bizkaia Spain

**Keywords:** Liver fibrosis, Hepatotoxicity

## Abstract

Iron overload and cellular senescence have been implicated in liver fibrosis, but their possible mechanistic connection has not been explored. To address this, we have delved into the role of iron and senescence in an experimental model of chronic liver injury, analyzing whether an iron chelator would prevent liver fibrosis by decreasing hepatocyte senescence. The model of carbon tetrachloride (CCl_4_) in mice was used as an experimental model of liver fibrosis. Results demonstrated that during the progression of liver fibrosis, accumulation of iron occurs, concomitant with the appearance of fibrotic areas and cells undergoing senescence. Isolated parenchymal hepatocytes from CCl_4_-treated mice present a gene transcriptomic signature compatible with iron accumulation and senescence, which correlates with induction of Reactive Oxygen Species (ROS)-related genes, activation of the Transforming Growth Factor-beta (TGF-β) pathway and inhibition of oxidative metabolism. Analysis of the iron-related gene signature in a published single-cell RNA-seq dataset from CCl_4_-treated livers showed iron accumulation correlating with senescence in other non-parenchymal liver cells. Treatment with deferiprone, an iron chelator, attenuated iron accumulation, fibrosis and senescence, concomitant with relevant changes in the senescent-associated secretome (SASP), which switched toward a more anti-inflammatory profile of cytokines. In vitro experiments in human hepatocyte HH4 cells demonstrated that iron accumulates in response to a senescence-inducing reagent, doxorubicin, being deferiprone able to prevent senescence and SASP, attenuating growth arrest and cell death. However, deferiprone did not significantly affect senescence induced by two different agents (doxorubicin and deoxycholic acid) or activation markers in human hepatic stellate LX-2 cells. Transcriptomic data from patients with different etiologies demonstrated the relevance of iron accumulation in the progression of liver chronic damage and fibrosis, correlating with a SASP-related gene signature and pivotal hallmarks of fibrotic changes. Altogether, our study establishes iron accumulation as a clinically exploitable driver to attenuate pathological senescence in hepatocytes.

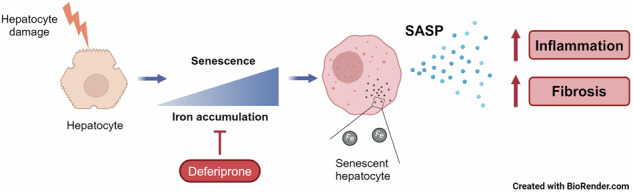

## Introduction

Chronic liver injury of various etiologies leads to fibrosis, which may progress toward cirrhosis and carcinogenesis [1]. The process occurs through a combination of different events: hepatocyte damage [2], inflammation [3], and activation of the hepatic stellate cells (HSC) into myofibroblasts, the main producers of extracellular matrix (ECM) proteins [4, 5]. Accumulating data derived from clinical and experimental studies have indicated that cellular senescence plays a relevant role in the occurrence and development of liver disease [6]. However, depending on the cells that become senescent, the results may differ. On one side, senescent hepatocytes accumulate in chronic liver diseases and create a pro-inflammatory environment as part of the senescence-associated secretory phenotype (SASP), which activates HSCs [7, 8] and impairs liver regeneration [9, 10], contributing to fibrosis progression. On the other side, senescence of myofibroblasts decreases the secretion of ECM, increases the expression of ECM-degrading enzymes, enhances immune surveillance, and, consequently, reduces liver fibrosis in mice [11]. Indeed, the design of specific senolytic agents that would act preferentially on hepatocytes but not on HSCs might represent a new therapeutic tool in liver fibrosis.

Different studies have shown that iron overload is associated with liver pathologies, triggering oxidative stress and mitochondrial dysfunction. The clearest example is the accumulation of iron in hepatocytes in hereditary hemochromatosis [12], but iron overload may also be a risk factor during the development and progression of metabolic diseases, such as metabolic dysfunction-associated steatotic liver disease (MASLD) [13], or in alcohol-associated liver disease (ALD) [14]. Liver iron is a surrogate marker of severe fibrosis in chronic hepatitis C [15]. Mice lacking hepcidin, a central regulator of iron homeostasis produced by hepatocytes, develop chronic liver injury and liver fibrosis [16]. Moreover, it has been proposed that high levels of TGF-β mediate *BMP2* reduction, which contributes to hepcidin downregulation in patients with liver fibrosis [17]. Despite this evidence, additional work is necessary to better understand whether iron overload is a general characteristic of chronic liver diseases and which are the underlying mechanisms by which excess iron can facilitate fibrotic responses [18].

We have recently proposed that iron accumulation could play a role in senescence and fibrosis in the kidney and lung [19]. In the case of the liver, it has been proposed that iron loading of hepatocytes in hereditary hemochromatosis leads to impaired replication and senescence, correlating with hepatic fibrosis [20]. However, compelling evidence of a correlation between iron accumulation and senescence in other liver pathologies is currently lacking.

The aim of this study was to delve into the role of iron and senescence in an experimental model of chronic liver injury and analyze whether an iron chelator would prevent the progression of liver fibrosis by decreasing hepatocyte senescence.

## Materials and methods

### Animal model

Carbon tetrachloride (CCl_4_)-induced liver fibrosis: CCl_4_ (289116, Sigma-Aldrich, St. Louis, MO, USA) was diluted in ultrapure mineral oil (M5310, Sigma-Aldrich) and injected intraperitoneally twice a week for 4–8 weeks (480 mg/kg) into 8 weeks old male C57BL/6J mice (Supplementary Fig. 1). Experimental groups included animals from different breedings and were randomly allocated to the different groups before starting the experiment. For deferiprone treatments, 1 week after the first CCl_4_ injection, half of the animals of both mineral oil and CCl_4_ groups were treated with 1 mg/mL deferiprone (379409, Sigma-Aldrich) dissolved in the drinking water. Investigators’ blindness to group allocation during the experiment was not possible due to procedural reasons. Mice were sacrificed 3 days after the last injection, and liver samples were collected. The procedure complied with the EU Directive 2010/63/UE for animal experiments and the institution’s guidelines and was approved by the General Direction of Environment and Biodiversity, Government of Catalonia (#4589) and the Ethical Committee for Animal Experimentation at IDIBELL, with the number EST-FOR-90.01. We have followed the 3R recommendations: Replacement, Reduction and Refinement.

### Tissue histopathological analysis

For analysis of collagen deposition, 5 µm-cut paraffin-embedded tissue sections were dewaxed and rehydrated in an Autostainer XL (Leica Biosystems, Deer Park, IL, USA) and stained with Picro-Sirius Red (Picric acid 197378; Direct-Red 80 365548, Sigma-Aldrich). To detect iron accumulation, enhanced Perl’s Prussian Blue (EPPB) staining was performed as previously described [19]. To specifically identify activated myofibroblasts, anti-α-SMA primary antibody (ab5694, Abcam, Cambridge, UK) was diluted 1:50, incubated overnight at 4 °C and binding developed with the Vectastain ABC kit (PK-4001, Vector Laboratories, Burlingame, CA, USA). Nuclei were stained with hematoxylin solution (MHS32, Sigma-Aldrich) and mounted in DPX (100579, Sigma-Aldrich). p21 immunohistochemistry was done as previously described [19]. All preparations were scanned on a virtual slide scanner NanoZoomer 2.0 HT (Hamamatsu, Tokyo, Japan) at the Histopathology Facility of the Institute for Research in Biomedicine (IRB) (Barcelona, Spain). ImageJ analysis software v1.44o (National Institutes of Health, Bethesda, MD, USA). The percentage of p21-positive stained cells was quantified with QuPath software v0.4.4 [21]. Further details about the quantification of the results are in Supplementary Information and/or in each figure legend.

### RNA sequencing (RNA-seq) analysis in hepatocytes

Primary mouse hepatocytes were isolated as previously described [22], as detailed in Supplementary Information. Total RNA from *Mus musculus* was quantified by Qubit® RNA BR Assay kit (Thermo Fisher Scientific, Bremen, Germany), and the RNA integrity was estimated by using RNA 6000 Nano Bioanalyzer 2100 Assay (Agilent, Santa Clara, CA, USA). The RNA-seq libraries were prepared as detailed in Supplementary Information. RNA-seq reads were processed using fastp v0.21 [23], including adapter removal, trimming of low-quality reads (Q < 30) and removal of reads with undetermined bases. Afterward, processed reads were aligned against the *Mus musculus* reference genome using GENCODE release M32 (GRCm39) using STAR v2.7.9 [24], and quantification of aligned reads to TPM was done with RSEM v1.3.1 [25]. Gene expression related to iron accumulation was analyzed with a gene signature previously designed by Maus et al. [19]. Analysis of a SASP-related gene expression was analyzed using two different gene signatures: Reactome and SenMayo [26]. Other gene signatures obtained from public databases are detailed in Supplementary Information.

### In vitro cell culture models

HH4 non-transformed human hepatocyte cell line was created in the Dr. Fausto lab by the introduction of the HPV E6 and E7 genes into human hepatocytes isolated from a normal adult liver, followed by the derivation of a clonal, immortalized cell line [27]. The hepatic stellate cell line LX-2 was kindly provided by Dr. Fouassier (CRSA, Paris, France) and authenticated before submission of this article. Cell lines were periodically tested for *Mycoplasma* to ensure contamination-free cultures. Cell viability was analyzed with crystal violet staining as previously described [28]. Culture conditions and treatments are mentioned in the text and detailed in Supplementary Information.

### Statistical analysis

To estimate sample size, dosage and treatment length, pilot experiments were used. All used animals were included in the analyses. For in vitro studies, at least three independent experiments were conducted in each case. In figures, data is expressed as mean (SD), and each dot represents an animal or sample, except when a representative experiment among three is shown. Statistical analysis was performed using GraphPad Prism v5.0 (GraphPad Software, Boston, MA, USA). When comparing two groups, two-tailed Mann–Whitney *U*-test was used. For multiple comparisons, one-way ANOVA was used with Sidak’s correction. Differences were considered statistically significant when *p* < 0.05.

Other methods are detailed in Supplementary Information.

## Results

### CCl_4_-induced liver fibrosis in mice is accompanied by iron accumulation and senescence in hepatocytes

**Fig. 1:**
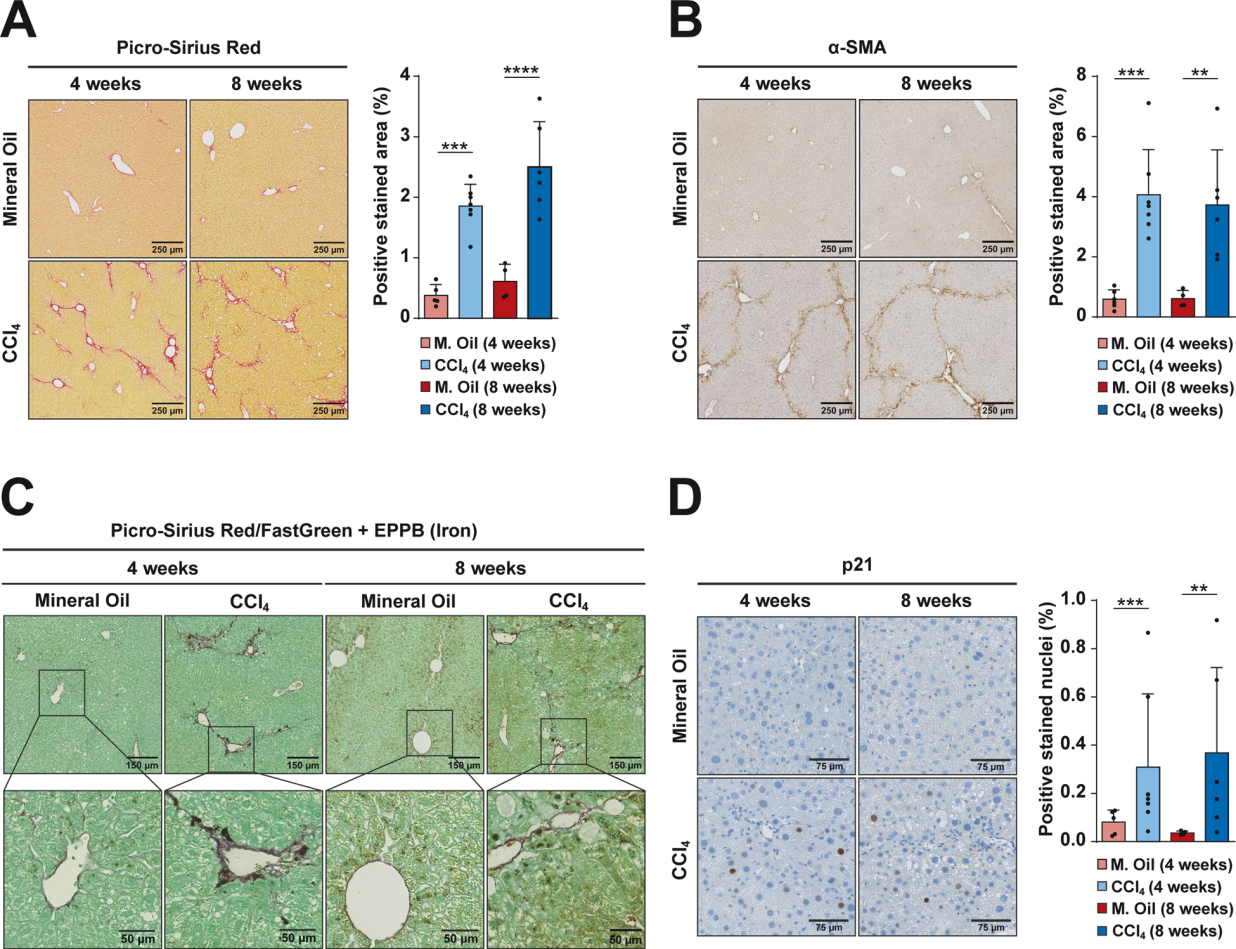
Characterization of fibrosis, iron accumulation and senescence in a CCl_4_-induced liver fibrosis murine model at 4 and 8 weeks. **A** Picro-Sirius Red staining was conducted to reveal collagen deposition. **B** Immunostaining of α-SMA as a marker of myofibroblasts. **C** To simultaneously detect fibrotic areas and iron accumulation, Picro-Sirius Red/FastGreen staining was combined with enhanced Perl’s Prussian Blue (EPPB). **D** Immunohistochemistry for the senescent marker p21. Images of representative areas are shown. In (**A** and **B**), the percentage of positive stained area was calculated using ImageJ software. Each dot represents the arithmetic mean of the percentage of positive stained area from three different regions for each animal. In (**D**), the percentage of positive nuclei was calculated using QuPath software. Each dot represents the percentage of positive nuclei quantified in a whole liver lobe for each animal. Data are presented as mean (SD) for each group. Number of animals per group is as follows: M. Oil 4 weeks (5), CCl_4_ 4 weeks (7), M. Oil 8 weeks (4), CCl_4_ 8 weeks (6). Statistical analysis was done using GraphPad Prism software (two-tailed Mann–Whitney *U*-test). *p < 0.05; **p < 0.01; ***p < 0.001; ****p < 0.0001.

**Fig. 2:**
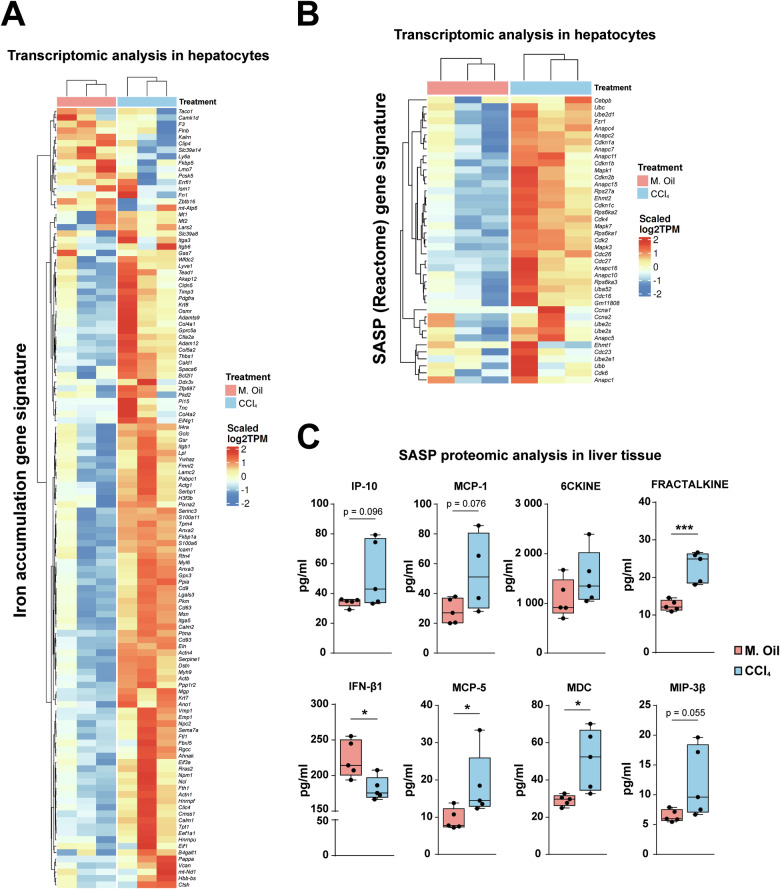
Analysis of the iron accumulation and SASP gene signatures in hepatocytes and proteomic analysis of SASP in liver tissues in the CCl_4_-induced liver fibrosis murine model. RNA-seq analysis was performed in hepatocytes isolated from mice treated with CCl_4_ or mineral oil (M. Oil) for 4 weeks (*n* = 3 mice/group). **A** Heatmap showing changes in gene expression for an iron accumulation gene signature (designed by Maus et al. [19]) in hepatocytes from CCl_4_-treated mice compared to mineral oil. **B** Heatmap showing changes in gene expression of the SASP gene signature from the Reactome collection in the same samples. **C** Liver tissue was collected from mice treated with CCl_4_ or mineral oil for 4 weeks, and a panel of 45 SASP-related factors was quantified at the protein level (*n* = 5 mice/group). A selection of some cytokines relevant to fibrosis is shown. Statistical analysis was done with two-tailed Mann–Whitney *U*-test. *p < 0.05.

Liver fibrosis in mice was induced by CCl_4_ chronic treatment, as described in “Materials and methods” and schematized in Supplementary Fig. 1. Collagen fibers, analyzed by Picro-Sirius Red, and the appearance of α-SMA positive areas, indicating activated myofibroblasts, were observed after 4–8 weeks of treatment (Fig. 1A, B). At both treatment times, deposits of iron were observed, close to the fibrotic areas (Fig. 1C), as well as some positive cells for p21, a hallmark of senescence (Fig. 1D). Importantly, a positive correlation between iron content and p21 was observed after 4 weeks of CCl_4_ treatment (Supplementary Fig. 2). At this time point, parenchymal cells (hepatocytes) were isolated and submitted to transcriptomic analysis by RNA-seq. A significant change in the expression of genes related to an iron accumulation signature, designed by Maus et al*.* [19], was observed (Fig. 2A), which correlated with the induction of ROS-related genes and activation of the TGF-β pathway (Supplementary Fig. 3A, B), as hallmarks of hepatocyte damage and a liver fibrotic process. The analysis of a SASP-related gene expression, which was analyzed using two different gene signatures that do not have any gene in common in mouse, revealed an increased expression in CCl_4_-treated livers (Fig. 2B and Supplementary Fig. 3C). SASP-related analysis at the protein level in the liver tissues revealed an increase in some proteins that are regulators of immune cell fate, such as chemokines involved in the recruitment and stimulation of monocytes (IP-10, MCP-1, MCP-5), or leukocytes (MDC, Fractalkine) (Fig. 2C), which reflected a role for fibrotic/senescent hepatocytes in regulating inflammation and immune microenvironment. Hepatocytes isolated from the CCl_4_-treated mice also showed strong changes in metabolism-related gene expression, particularly oxidative phosphorylation and fatty acid oxidation pathways (Supplementary Fig. 4A, B). Overall, the activity of these pathways decreased coincident with the increase in iron accumulation and SASP pathways (Supplementary Fig. 4C).

Using previously published transcriptomic data from hepatocytes treated in vitro with CCl_4_ [29], we observed that iron accumulates after 3 days of treatment, concomitant with an increase in SASP, ROS and TGF-β gene transcriptomic pathways (Supplementary Fig. 5A). Analysis of the iron-related gene signature in a published single-cell RNA-seq dataset from CCl_4_-treated livers [30] indicated that iron also accumulates in other non-parenchymal liver cell types, significantly correlating with senescence in HSC, endothelial cells (EC) and cholangiocytes (Chol) (Supplementary Fig. 6). Interestingly, using previous bulk transcriptomic data from chronic CCl_4_-treated livers [31], it may be observed that iron appears to accumulate during the progression of liver fibrosis, concomitant with an increase in SASP, TGF-β and fibrotic gene transcriptomic pathways, but all these pathways decreased at longer times, in the tolerance phase (Supplementary Fig. 5B).

### Treatment with deferiprone attenuates iron accumulation, fibrosis and senescence in CCl_4_-treated mice

**Fig. 3:**
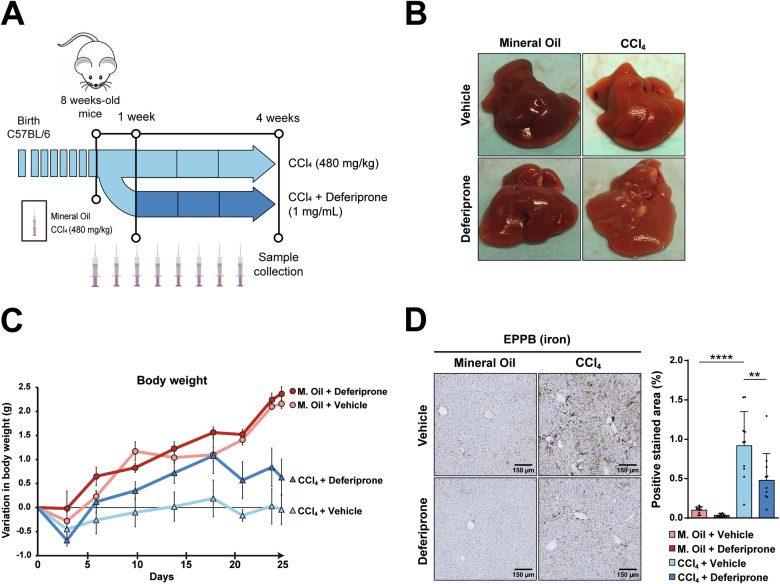
Effects of deferiprone on iron accumulation in the model of CCl_4_-induced liver fibrosis. **A** Schematic representation of the experimental design to induce liver fibrosis by intraperitoneal injections of CCl_4_ (480 mg/kg) or mineral oil (M. Oil) twice a week for 4 weeks and treatment without or with deferiprone 1 mg/mL in the drinking water. **B** Representative images of the livers from each group at sacrifice. **C** Variation in body weight along the experiment. **D** Iron accumulation analyzed by enhanced Perl’s Prussian Blue (EPPB) staining in paraffin-embedded liver sections. Representative areas are shown for each group. Percentage of positive stained area was quantified for each animal using ImageJ software. Each dot represents the arithmetic mean of the percentage of positive stained area from three different regions for each animal. Data are presented as mean (SD) (*n* = 10 mice/group). Statistical analysis was done with one-way ANOVA with Sidak’s correction. *p < 0.05; **p < 0.01; ***p < 0.001; ****p < 0.0001.

To analyze the relevance of iron accumulation in the fibrosis progression, as well as its relevance in senescence, mice were treated with deferiprone from 1 week after the first CCl_4_ injection, as described in “Materials and methods” and schematized in Fig. 3A, accompanied by representative images of the livers (Fig. 3B). The variation in body weight along the experiment (Fig. 3C) showed a better progression in the group of mice that were injected with CCl_4_ and received deferiprone, compared with CCl_4_ alone, suggesting a potential therapeutic effect. The iron deposits found in the mice receiving CCl_4_ decreased significantly by the treatment with deferiprone (Fig. 3D). Expression of the transferrin receptors 1 and 2 (*Tfr1/2*) and the homeostatic iron regulator (*Hfe)*, which regulates the binding and endocytosis of the circulating Fe-transferrin into the cell, were found upregulated in livers from mice treated with deferiprone, indicating a response of the cells in a situation of decreased iron levels (Supplementary Fig. 7). It is worth mentioning that changes were small, which evidence that the dose of deferiprone used was not causing a systemic iron deficiency.

**Fig. 4:**
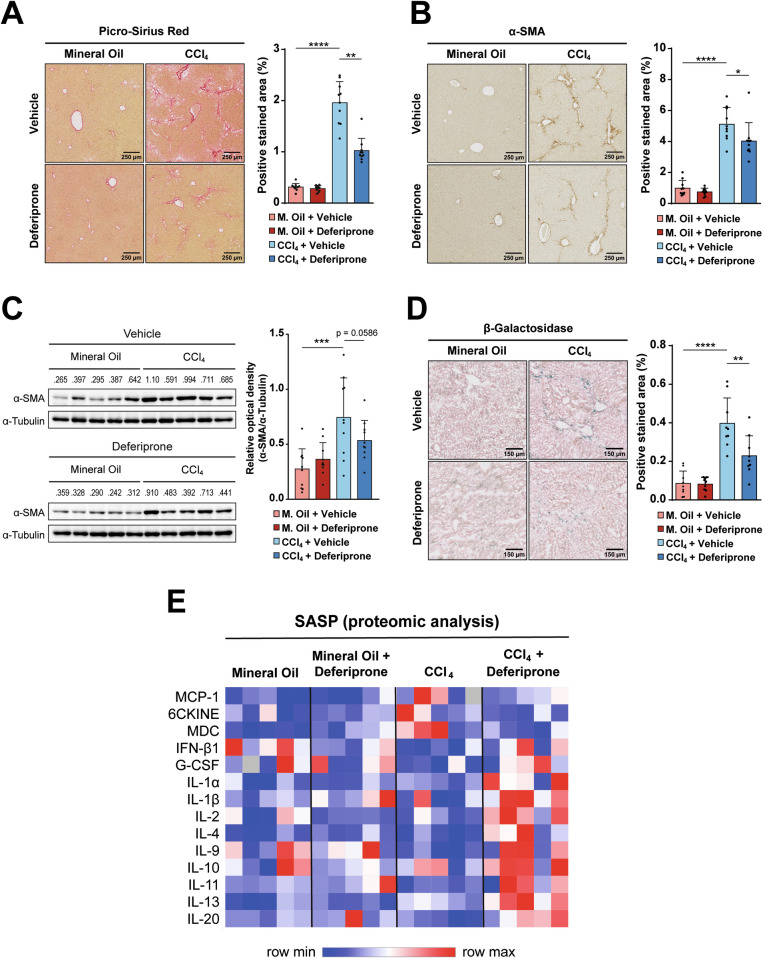
Effects of deferiprone on fibrosis and senescence hallmarks in the mouse CCl_4_-induced liver fibrosis model. Fibrosis was analyzed in paraffin-embedded tissue sections after 4 weeks of CCl_4_ treatment: **A** Picro-Sirius Red staining and **B** α-SMA immunohistochemistry as a marker of myofibroblasts. **C** Western blot analysis of α-SMA protein levels in the liver tissue. **D** Fresh liver tissues embedded in OCT were cut in sections and stained for detection of β-Galactosidase activity. For (**A**–**C**), images of representative areas are shown for each group. Percentage of positive stained area was quantified for each animal using ImageJ software. Each dot represents the arithmetic mean of the percentage of positive stained area from three different regions for each animal. Results are shown as mean (SD) (*n* = 10 mice/group). Two-tailed Mann–Whitney *U*-test was used for statistical analysis. *p < 0,05; **p < 0.01; ***p < 0.001; ****p < 0.0001. **E** A panel of 45 cytokines and chemokines characteristic of the SASP were analyzed at the protein level in tissue samples, *n* = 5 mice/group. Data were analyzed carefully, and those factors with statistically significant changes (one-way ANOVA with Sidak’s post-hoc test, *p*-value < 0.05) are summarized in a heatmap format.

Having confirmed that deferiprone was preventing iron accumulation upon CCl_4_ treatment, we analyzed the effects on fibrosis. The fibrotic collagen area, as stained by Picro-Sirius Red, was decreased in the mice treated with deferiprone compared to CCl_4_ alone (Fig. 4A). Analysis of the α-SMA, as a myofibroblastic marker, showed similar results, with a significant decrease when analyzed by IHC (Fig. 4B) and corroborated by western blot (Fig. 4C and Supplementary uncropped blots). These results indicated that deferiprone attenuates liver fibrosis induced by CCl_4_ in mice.

Then, we moved to analyze the hallmarks of senescence. As seen in Fig. 4D, the staining for β-Galactosidase activity revealed the presence of senescent cells around the fibrotic area in the CCl_4_ group, and importantly, deferiprone treatment significantly decreased the percentage of senescent area. Analysis of SASP-related proteins revealed that CCl_4_ treatment induced the production of inflammatory cytokines such as MCP-1, 6CKINE or MDC. This cytokine regulation pattern was not found in animals treated with deferiprone (Fig. 4E). Moreover, antifibrotic or anti-inflammatory cytokines, such as IFN-β1, IL-4, IL-9, IL-10, IL-13 or IL-20, were significantly increased in the CCl_4_+Deferiprone group compared to CCl_4_ alone. IL-2, a cytokine that mediates the specific expansion of Tregs, which may ameliorate tissue damage following CCl_4_ administration [32], also presented higher levels in the CCl_4_+Deferiprone group.

### Deferiprone attenuates the senescent phenotype induced by doxorubicin in human hepatocytes, but not in HSC

**Fig. 5:**
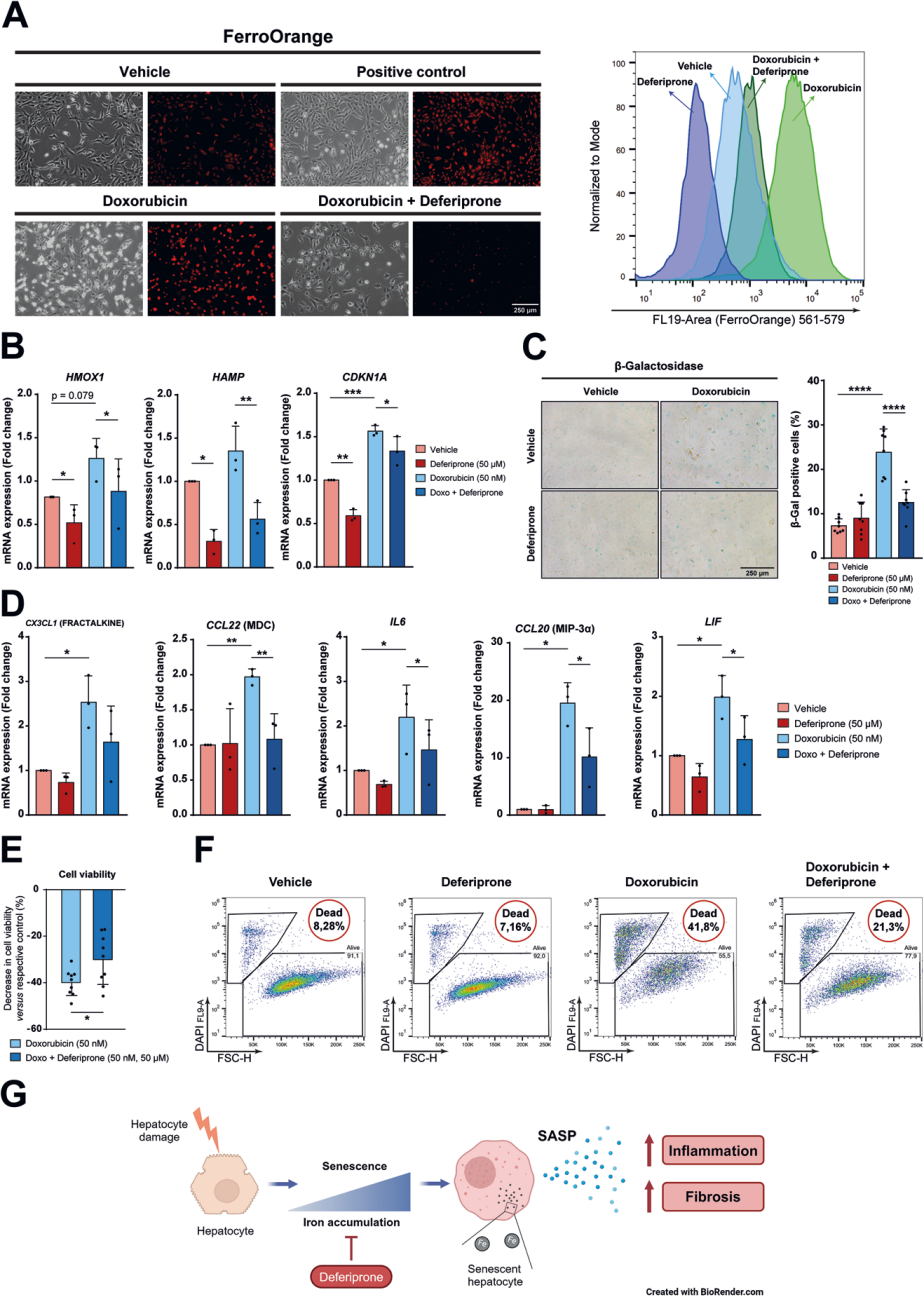
Effects of deferiprone on iron accumulation, senescence and cell viability in human hepatocytes. The human hepatocyte cell line HH4 was cultured as described in Supplementary Fig. 8A. **A** FerroOrange dye was used to analyze iron accumulation. Treatment for 24 h with iron (660 µM) was used as positive control. Representative images were taken with a fluorescence microscope (left) and quantification was performed by flow cytometry (right). **B** Gene expression of *HMOX1* and *HAMP* as relevant genes related to iron metabolism and *CDKN1A* as a marker of senescence were analyzed by RT-qPCR. **C** β-Galactosidase staining assay was performed to analyze senescence. At least 10 images were taken from random fields by phase contrast microscopy (left) and percentage of positive cells was quantified (right). **D** RT-qPCR of a panel of SASP-related genes (*CX3CL1, CCL22, IL6, CCL20* and *LIF*). **E** Cell viability was assessed by crystal violet and expressed as loss of viability *versus* the respective control (vehicle or deferiprone). **F** Flow cytometry with DAPI staining was used to determine the percentage of dead cells upon 6 days of treatments. **G** Diagram summarizing the message of the manuscript in a visual way. Briefly, iron accumulation during chronic liver damage induces senescence and SASP in hepatocytes, which would contribute to liver inflammation and fibrosis, and these hallmarks can be attenuated by deferiprone. All analyses in panels (**A**–**F**) were done on day 6 post-treatment. Statistical analysis was done with one-way ANOVA with Sidak’s correction (**B**) or two-tailed Mann–Whitney *U*-test (**C**–**E**) (*n* = 3 independent experiments). In (**A**, **C** and **F**), a representative experiment is shown. *p < 0.05; **p < 0.01; ***p < 0.001; ****p < 0.0001.

We next wondered whether deferiprone could prevent senescence in a human hepatocyte cell line (HH4 cells) in vitro. We used doxorubicin (50 nM) to induce senescence alone or in co-treatment with deferiprone (50 µM), as detailed in “Materials and methods” section and schematized in Supplementary Fig. 8A. As expected [19], doxorubicin increased the intracellular labile iron content, which was attenuated by deferiprone (Fig. 5A). In the presence of deferiprone, a decrease in the expression of iron-induced genes, such as *HMOX1* or *HAMP*, was seen (Fig. 5B). The increase in β-Galactosidase observed after doxorubicin treatment in hepatocytes was almost completely abolished by deferiprone (Fig. 5C), which also slightly attenuated the upregulation of *CDKN1A* (p21), another hallmark of senescence (Fig. 5B). The analysis of SASP-related gene expression revealed that doxorubicin induced the expression of *CX3CL1* (Fractalkine), *CCL22* (MDC), *IL6*, *CCL20* (MIP-3α) or *LIF*, which were clearly attenuated in doxorubicin and deferiprone combined treatment in hepatocytes (Fig. 5D). Furthermore, deferiprone significantly attenuated the decrease in cell number and cell viability produced by doxorubicin in hepatocytes (Fig. 5E, F). The appearance of apoptotic cells by doxorubicin (Fig. 5E) was not related to ferroptosis, as determined by a probe used to detect ferroptosis (BODIPY C11) (Supplementary Fig. 9A). Also, the in vivo transcriptomic analysis in hepatocytes did not support the induction of ferroptosis by CCl_4_ treatment (Supplementary Fig. 9B). Indeed, we propose that iron accumulation during chronic liver damage induces senescence and SASP in hepatocytes, which would contribute to liver inflammation and fibrosis (Fig. 5G).

**Fig. 6:**
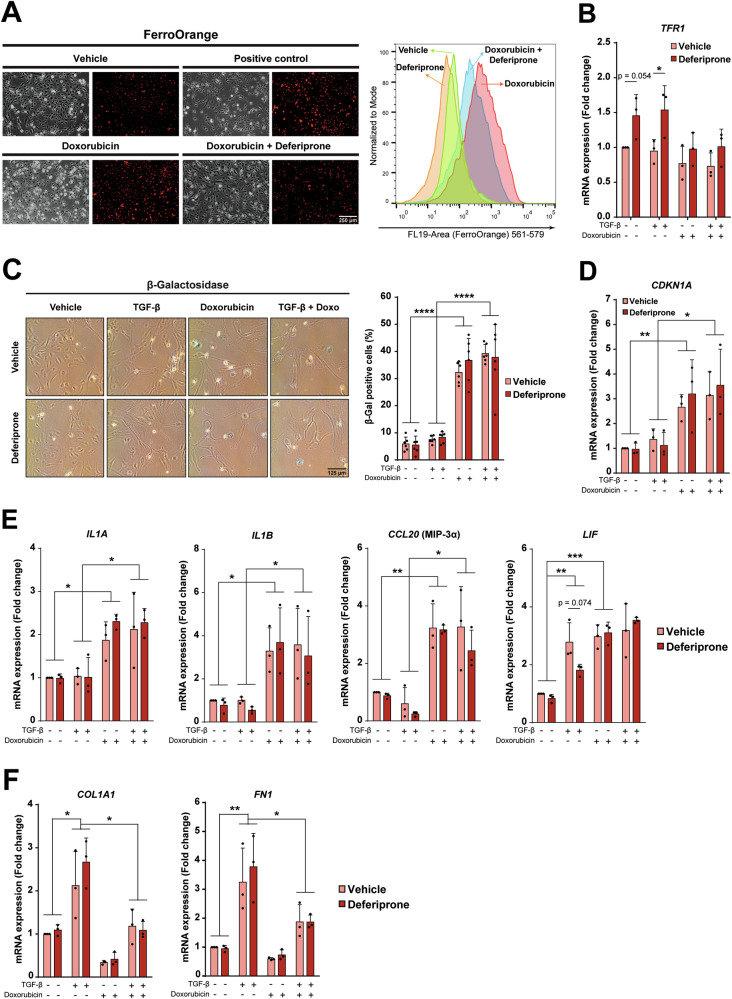
Effects of deferiprone on iron accumulation, senescence and activation markers in human HSC. The human HSC cell line LX-2 was cultured, as detailed in Supplementary Fig. 8B, with doxorubicin as a senescent inducer. **A** FerroOrange dye was used to analyze iron accumulation. Treatment for 24 h with iron (660 µM) was used as positive control. Representative images were taken with a fluorescence microscope (left) and quantification was performed by flow cytometry (right). **B** *TFR1* mRNA levels were analyzed by RT-qPCR. **C** β-Galactosidase staining assay was performed to analyze senescence. Six images were taken from random fields by phase contrast microscopy (left) and percentage of positive cells was quantified (right). **D**–**F** Analysis of mRNA levels by RT-qPCR of *CDKN1A*, a panel of SASP-related genes (*IL1A, IL1B, CCL20* and *LIF*) and HSC activation-related genes (*COL1A1, FN1*). All analyses in panels (**A**–**F**) were done on day 6 post-treatment. Statistical analysis was done with one-way ANOVA with Sidak’s correction (*n* = 3 independent experiments). In (**A** and **C**), a representative experiment is shown *p < 0.05; **p < 0.01; ***p < 0.001; ****p < 0.0001.

Then, we checked whether iron chelators could also modulate senescence and/or profibrotic activity (in response to TGF-β) in HSC, by using the LX-2 cell line (Supplementary Fig. 8B). An increase in iron accumulation by doxorubicin was also observed (Fig. 6A). Deferiprone attenuated iron accumulation and upregulated the expression of *TFR1* (Fig. 6B), which increases in situations of decreased iron availability. However, the increase in the percentage of β-Gal-positive cells produced by doxorubicin was not altered by the presence of deferiprone (Fig. 6C), correlating with no effects on *CDKN1A* upregulation (Fig. 6D). The analysis of SASP-related gene expression revealed that doxorubicin induced the expression of *IL1A, IL1B*, *CCL20* (MIP-3α) or *LIF* (which also showed upregulation by TGF-β). However, deferiprone was not able to attenuate the doxorubicin-induced SASP-related gene expression in hepatic stellate cells (Fig. 6E). As previously suggested [11], senescence induced by doxorubicin attenuated the TGF-β-mediated activation of HSC, as evidenced by the decreased expression of *COL1A1* or *FN1*, regardless of whether or not deferiprone was present (Fig. 6F). To corroborate these results, we next used an alternative pro-senescent factor in HSC, to demonstrate whether deferiprone does not produce any effect, neither. We used deoxycholic acid (DCA), as previously described in the literature, as a senescence inducer in HSC [33]. Results demonstrated similar results to those obtained with doxorubicin (Supplementary Fig. 10). Identical results were also observed using higher concentrations of deferiprone (50 µM) in both models (results not shown).

Altogether, these results indicate that deferiprone counteracts senescence specifically in hepatocytes and has no effects on senescent HSC.

### Transcriptomic analyses of human samples reveal an increase in iron accumulation-related gene signature during the progression of fibrosis, correlating with a SASP gene signature

**Fig. 7:**
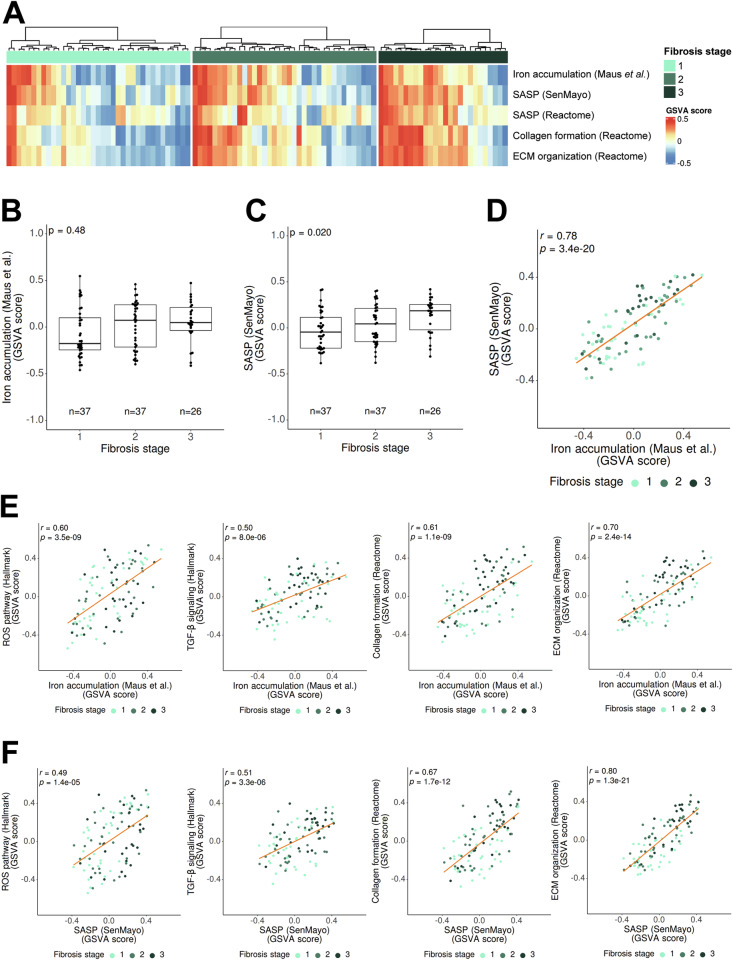
Analysis of iron accumulation and SASP expression across liver fibrosis stages in human patients. **A** Heatmap showing changes in gene expression for the following gene signatures: iron accumulation (designed by Maus et al. [19]), SASP (SenMayo and Reactome), collagen formation (Reactome) and ECM organization (Reactome) in the Fujiwara et al. cohort of liver fibrosis patients [34], classified by fibrosis stage. **B** Boxplot of relative enrichment (GSVA score) for iron accumulation gene signature across fibrosis stages. **C** Boxplot of SASP (SenMayo) gene signature relative enrichment (GSVA score) across fibrosis stages. **D** Pearson correlation analysis of the relative enrichment of iron accumulation gene signature with SASP (SenMayo) gene signature. **E**, **F** Pearson correlation analyses of the relative enrichment in the gene signatures related to iron accumulation (**D**) and relative enrichment of SASP (SenMayo) (**E**) with ROS pathway (Hallmark), TGF-β signaling (Hallmark), collagen formation (Reactome) and ECM organization (Reactome) gene signatures. Each dot is a sample (color indicates the fibrosis stage). Kendall’s τ was used to assess the association between the gene signatures and fibrosis stage. All analyses were adjusted for multiple testing with Bonferroni test correction.

Analysis of public data using molecular signatures from non-alcoholic fatty liver disease (now named MASLD) [34] demonstrated that the iron accumulation gene transcriptomic signature showed a tendency to increase, and the SASP-related gene expression signature significantly increased along the stages of liver fibrosis (Fig. 7A–C). A strong correlation between iron accumulation and SASP (SenMayo) gene signatures were observed (Fig. 7D). No increase in the ferroptosis-related gene signature was observed along the fibrosis stages and correlations between ferroptosis and SASP gene signatures were weak and observed with only one of the SASP gene signatures (Supplementary Fig. 11). Both iron accumulation and SASP gene signatures correlated with ROS-related genes, activation of the TGF-β pathway, collagen formation and ECM organization gene signatures (Fig. 7E, F). To further validate these findings, we performed the same analysis with the second SASP gene signature (Reactome), obtaining identical results (Supplementary Fig. 12).

**Fig. 8:**
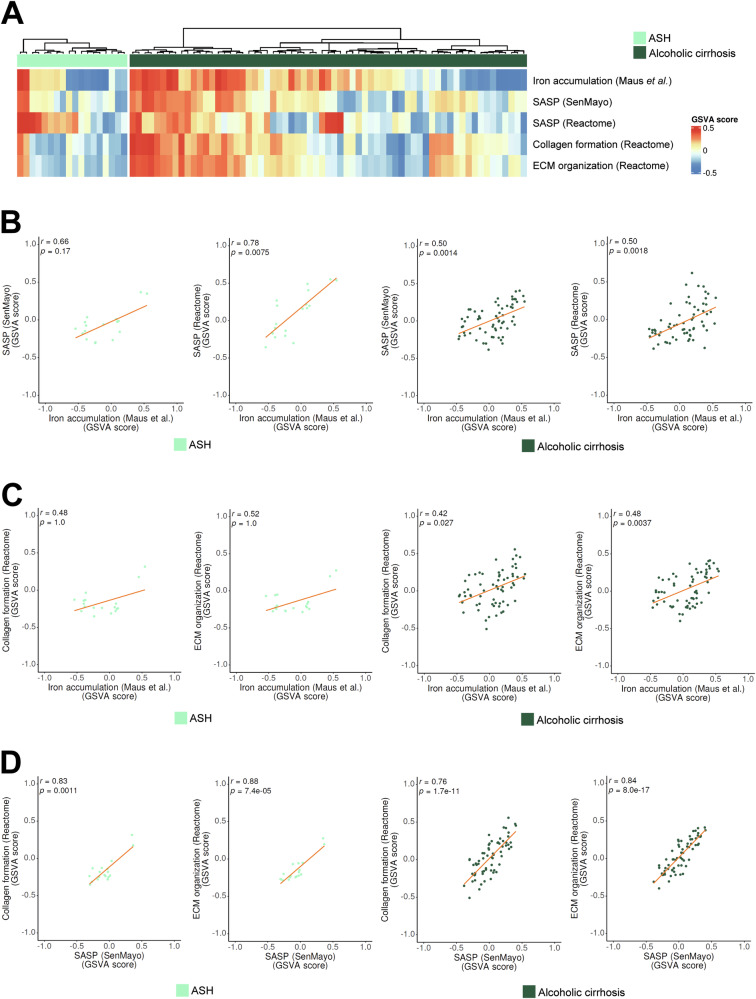
Analysis of iron accumulation and SASP expression in HCC-naïve ASH and alcoholic cirrhosis patients. **A** Heatmap showing changes in gene expression for the following gene signatures: iron accumulation (designed by Maus et al. [19]), SASP (SenMayo and Reactome), collagen formation (Reactome) and ECM organization (Reactome) in the Trepo et al. cohort of HCC-naïve ASH and alcoholic cirrhosis patients [35]. **B** Pearson correlation analysis of the relative enrichment of iron accumulation gene signature with SASP (SenMayo and Reactome) gene signatures in ASH (left) and alcoholic cirrhosis (right) patients. **C**, **D** Pearson correlation analyses of the relative enrichment in the gene signatures related to iron accumulation (**C**) and relative enrichment of SASP (SenMayo) (**D**) with collagen formation (Reactome) and ECM organization (Reactome) gene signatures in ASH (left) and alcoholic cirrhosis (right) patients. Each dot is a sample. All analyses were adjusted for multiple testing with Bonferroni test correction.

We also explored public transcriptomic data from another cohort of patients suffering alcoholic steatohepatitis (ASH) and alcoholic cirrhosis [35]. Although some heterogeneity among the patients was observed (Fig. 8A), a clear positive correlation was patent between iron accumulation and SASP, collagen formation and ECM pathways (Fig. 8B, C), particularly in the cirrhotic patients. A strong significant correlation was also observed between SASP and collagen formation/ECM pathways (Fig. 8D).

These analyses support the clinical relevance of iron accumulation in human liver fibrotic pathologies, establishing a strong correlation with pathological senescence and hallmarks of liver fibrosis.

## Discussion

Despite extensive knowledge of liver fibrosis mechanisms, antifibrotic therapies in human pathologies have shown modest success [36, 37]. Better refined and more predictive in vitro and animal models may hasten drug development [38]. An increasing number of studies on hepatocellular senescence have revealed its importance in liver physiology and pathology [6, 39]. However, the mechanisms that induce hepatic senescence are yet to be fully explored. Here, we show that senescence occurs in an experimental model of liver fibrosis after chronic treatment with CCl_4_, concomitant with iron accumulation and hallmarks of liver fibrosis. These results reveal that not only the pathologies related to MASLD or ALD accumulate iron [13, 14], but it may also accumulate in cases of chronic damage of the hepatocytes, not related to steatohepatitis. Interestingly, using previous bulk transcriptomic data from chronic CCl_4_-treated livers [31], we demonstrate that iron accumulates during the progression of liver fibrosis, but decreases in the tolerance phase that has been suggested to be the active auto protection program of the liver when harmed repeatedly [31].

The treatment with an iron chelator, deferiprone, attenuates iron accumulation and fibrosis, revealing the essential role played by iron in this process. Some previous evidence indicated that deferoxamine, an iron chelator, could be antifibrotic in liver, which authors related to its antioxidant properties [40, 41]. Here we propose that deferiprone prevents liver fibrosis progression by decreasing senescence. Senescent cells are highly secretory, and they execute a diverse set of functions mediated by SASP [42]. Results shown here indicate that deferiprone treatment induces notable changes in the composition of SASP in the liver, favoring an anti-inflammatory environment.

The major contributors to senescence properties in the liver are hepatocytes [39]. Hepatocytes express almost all the iron-related genes, in accordance with their central role in iron metabolism [43]. Here, we present evidence that hepatocytes from CCl_4_-treated mice, or treated in vitro with CCl_4_, show a gene transcriptomic signature compatible with iron accumulation and SASP, correlating with induction of ROS-related genes, activation of the TGF-β pathway and a decrease in oxidative metabolism, as characteristics of hepatocyte growth arrest, inflammation and senescence [44]. The results in in vitro experiments with human hepatocytes have demonstrated that iron accumulates in response to an agent that induces senescence, such as doxorubicin, being deferiprone able to attenuate some senescent-related hallmarks, such as SA-β-GAL staining and the SASP-related gene expression. Overall, no doubt that hepatocytes would be a target for iron chelators, which would prevent senescence. Deferiprone also attenuated doxorubicin-mediated cell death but did not completely stop the arrest in the cell cycle, as observed by the levels of *CDKN1A* (p21), which continue to be high, and the cell viability, which continues to be lower, in the combined treatment with doxorubicin and deferiprone. This type of phenotype is typical of treatments that impair the SASP in senescent cells, although are not able to completely prevent the arrest in the cell cycle. However, this SASP-less phenotype is sufficient to attenuate the microinflammatory involvement that contributes to liver fibrosis and cancer [45, 46].

The use of senolytic agents in liver fibrosis could be used to remove senescent hepatocytes, but action on HSC could be deleterious [47]. Different strategies are ongoing to target senescence, specifically in hepatocytes [48]. Here, we demonstrate that iron chelators would prevent the deleterious effects of iron accumulation and the progression of senescence, specifically in hepatocytes, preventing inflammation and fibrosis. No effect of deferiprone is observed in HSC undergoing senescence by doxorubicin or DCA and it does not affect their expression of ECM-related genes.

Altogether, experimental data indicate that iron, which accumulates after chronic liver injury, mediates senescence and liver fibrosis. We also show transcriptomic data in patients with different etiologies (MASH, ASH and alcoholic cirrhosis) demonstrating the relevance of iron accumulation in the progression of liver fibrosis, correlating with a SASP-related gene signature and pivotal hallmarks of hepatocyte damage. Collectively, our study establishes iron accumulation as a clinically exploitable driver of pathological senescence in hepatocytes, which could attenuate liver fibrosis, as was previously proposed in kidney and lung [19].

## Supplementary information

Supplementary information

Supplementary uncropped western blots

Report for authentication LX-2 cells

## Data Availability

Raw and processed data from the RNA-seq analysis in hepatocytes have been submitted to Gene Expression Omnibus (GEO) and are publicly available under the code GSE265969. Other data are available on request from the corresponding author [IF].
