# Supplementary material for: Iron chelation as a new therapeutic approach to prevent senescence and liver fibrosis progression
Source: Cell Death Dis. 2024 Sep 17;15(9):680. doi: 10.1038/s41419-024-07063-0 (PMC11408630; doi:10.1038/s41419-024-07063-0)
Supplement: Supplementary file 1 — Supplementary information [file 41419_2024_7063_MOESM1_ESM.pdf]

Supplementary Information

Iron chelation as a new therapeutic approach to prevent senescence and liver fibrosis progression

Josep Amengual[1,2], Ania Alay[3,4], Javier Vaquero[1,2,5], Ester Gonzalez-Sanchez[1,2,5,6], Esther Bertran[1,2], Aránzazu Sánchez[7,8] , Blanca Herrera[7,8], Kathleen Meyer[9,10], Mate Maus[9,11], Manuel Serrano[9,10,12], María Luz Martínez-Chantar[2,13], Isabel Fabregat[1,2#]

[1]TGF-β and Cancer Group. Oncobell Program, Bellvitge Biomedical Research Institute (IDIBELL), L'Hospitalet de Llobregat, Barcelona, Spain.

[2]Centro de Investigación Biomédica en Red de Enfermedades Hepáticas y Digestivas (CIBERehd), Madrid, Spain.

[3]Unit of Bioinformatics for Precision Oncology, Catalan Institute of Oncology (ICO), L'Hospitalet de Llobregat, Barcelona, Spain.

[4]Preclinical and Experimental Research in Thoracic Tumors (PReTT), Oncobell Program, IDIBELL, L'Hospitalet de Llobregat, Spain.

[5]HepatoBiliary Tumours Lab, Centro de Investigación del Cáncer and Instituto de Biología Molecular y Celular del Cáncer, CSIC-Universidad de Salamanca, Salamanca 37007, Spain.

[6]Department of Physiology and Pharmacology, University of Salamanca, 37007, Salamanca, Spain

[7]Department of Biochemistry and Molecular Biology, Faculty of Pharmacy, Complutense University of Madrid, Madrid, Spain.

[8]Health Research Institute of the "Hospital Clínico San Carlos" (IdISSC), Madrid, Spain.

[9]Institute for Research in Biomedicine (IRB Barcelona), The Barcelona Institute of Science and Technology (BIST), Barcelona, Spain.

[10]Altos Labs, Cambridge Institute of Science, Cambridge, United Kingdom.

[11]Vall d'Hebron Institute of Oncology, Barcelona, Spain.

[12]Catalan Institution for Research and Advanced Studies (ICREA), Barcelona, Spain.

[13]Liver Disease and Liver Metabolism Laboratory, CIC bioGUNE-BRTA (Basque Research & Technology Alliance), Derio, Bizkaia, Spain.

#Corresponding author: Isabel Fabregat. Email: ifabregat@idibell.cat

Supplementary Information for the Material and Methods Section

Isolation of mouse hepatocytes and RNA-seq analysis

After 4 weeks of treatment (with mineral oil or CCl_4), livers were perfused with Hank's balanced salt solution supplemented with 10 mM Hepes and 0.2 mM EGTA for 5 min, followed by a 15 min perfusion with William's medium E containing 10 mM Hepes and 0.03% collagenase type 1 (125 U/mg; LS0041, Worthington Biochemical Corp, Lakewood, NJ, USA). Livers were further minced, filtered through a 70 µm cell strainer (BD Biosciences, Franklin Lakes, NJ, USA) and viable hepatocytes were selected by centrifugation in Percoll (17089101, Cytiva, Marlborough, MA, USA) and stored at -80 ºC.

The RNA-seq libraries were prepared with KAPA Stranded mRNA-Seq Illumina® Platforms Kit (Roche, Basel, Switzerland) following the manufacturer's recommendations starting with 500 ng of total RNA as the input material. The library was quality controlled on an Agilent 2100 Bioanalyzer with the DNA 7500 assay. The libraries were sequenced on NovaSeq 6000 (Illumina, San Diego, CA, USA) with a read length of 2x151 bp, following the manufacturer's protocol for dual indexing. Image analysis, base calling and quality scoring of the run were processed using the manufacturer's software Real Time Analysis (RTA v3.4.4).

Analysis of gene expression by RT-qPCR

mRNA expression levels of genes were analyzed by Real Time-quantitative PCR (RT-qPCR). Total RNA was isolated from the different cells or tissues using EZNA Total RNA Kit II (Omega Bio-tek, Norcross, GA, USA). cDNA was produced using the High-capacity cDNA Reverse Transcription Kit (Applied Biosystems, Waltham, MA, USA). RT-qPCR was performed in duplicate in a Light Cycler 480 II (Roche). SYBR Green PCR Master Mix was used for PCR reactions (Applied Biosystems). Primers are listed in ***Suppl. Table 1.*** *RPL32* gene was used as housekeeping.

Quantification of IHC results

Slide scans were examined thoroughly for analyses. In Picro-Sirius Red, α-SMA and EPPB preparations, three representative regions from different liver lobes of each animal were selected and the percentage of positive stained area was quantified with ImageJ analysis software v1.44o (National Institutes of Health, Bethesda, MD, USA). For each animal, the arithmetic mean of the stained area from the different selected regions was then

calculated. The percentage of p21-positive stained cells was quantified with QuPath software v0.4.4 (ref. 21 in main text) in a whole liver lobe for each animal. To ensure blinding when assessing the outcome in histological preparations, automatization of quantification by QuPath and predetermination of settings in ImageJ was performed.

Western blot analysis

To analyze protein levels by Western blot, liver tissue was lysed in RIPA lysis buffer supplemented with a cocktail of protease inhibitors (11697498001, Roche Diagnostics, Rotkreuz, Switzerland) and orthovanadate (S6508, Sigma) at 4 °C. Lysis was performed in a Tissue Lyser II (QIAGEN, Venlo, The Netherlands) and protein concentration was determined using a Pierce™ BCA protein assay kit (Thermo Fisher Scientific). Proteins were separated with denaturalizing SDS-polyacrylamide gel electrophoresis (SDS-PAGE) and transferred to a nitrocellulose membrane in wet conditions. For immunoblotting, membranes were incubated in 5% bovine serum albumin (A7906, Sigma) in PBS-Tween 0.05% for 1 h at room temperature. Afterwards, membranes were incubated with the primary antibodies against α-SMA (ab5694, Abcam) and α-Tubulin (T9026, Sigma) both at 1/1000 dilution, overnight at 4 °C. The following day primary antibodies were washed with PBS-Tween 0.05% and binding was developed with secondary antibodies diluted 1/2000 in PBS-Tween 0.05% (NA934V anti-Rabbit and NA931V anti-Mouse for α-SMA and α-Tubulin, respectively), incubated for 1 h at room temperature. Finally, positive hybridization was visualized with a chemiluminescent solution (GE HealthCare, Little Chalfont Amersham, UK) in a ChemiDoc™ Touch Imaging System (Bio-Rad, Munich, Germany) and densitometric analysis was performed using Image Lab™ software (Bio-Rad).

Analysis of the SASP in liver tissue

Liver tissues were lysed in RIPA buffer and centrifuged to separate insoluble debris. Afterwards, protein concentration was determined using a Pierce™ BCA protein assay kit (Thermo Fisher Scientific) and adjusted so that all samples contained the same amount of protein. Samples were shipped to an external commercial laboratory (Eve Technologies Corp, Calgary, Canada) and a Mouse Cytokine/Chemokine 44-Plex Discovery Assay® Array (MD44) was performed.

In vitro cell culture models

The hepatocyte HH4 cells were plated in MEM with 15% FBS and after 24 h, medium was changed to deplete FBS up to 2% FBS. 24 h later, treatments were initiated, and cells were

cultured for 6 days more. Treatments were as follows: vehicle; 50 nM doxorubicin (S1208, Selleck Chemicals, Cologne, Germany) to induce senescence; 50 μM deferiprone; and doxorubicin + deferiprone. Culture media and treatments were refreshed after 3 days.

The HSC LX-2 cells were cultured in DMEM with 10% FBS and 24 h later medium was changed to deplete FBS up to 0.5% FBS. The following day, cells were treated with vehicle, 2 ng/mL TGF-β (T7039, Sigma-Aldrich), 50 nM doxorubicin or 80 μM deoxycholic acid (DCA; D6750, Sigma-Aldrich) to induce senescence, or the corresponding senescent inducer combined with TGF-β, both in the absence or presence of 20 μM deferiprone and maintained for 6 days, refreshing the media and treatments after 3 days.

Concentration of deferiprone in each case was designed as the amount able to attenuate iron accumulation without serious effects on cell death. LX-2 cells were more sensitive to deleterious effects of deferiprone.

Detection of intracellular iron

Labile iron was measured using the FerroOrange probe (36104, Cell Signaling Technology, Danvers, MA, USA) following manufacturer's instructions. A 24 h treatment with an aqueous solution of 330 μM iron sulfate (F8633, Sigma-Aldrich) and 330 μM iron nitrate (F8508, Sigma-Aldrich) was used as positive control. Cells were examined through a Leica DM IRB Inverted Fluorescence Microscope (Leica Microsystems, Wetzlar, Germany). For analysis by flow cytometry, cells were dissociated and processed in a MoFlo Astrios Cell Sorter (Beckman Coulter, Brea, CA, USA) at the Biology-Bellvitge Unit from Scientific and Technological Centers (CCiTUB), Universitat de Barcelona.

SA-β-GAL staining

Cells were stained for senescence-associated β-galactosidase activity with a commercial kit (#9860, Cell Signaling Technology), following manufacturer's instructions. Staining of mouse liver tissue was done as stated in Maus *et al*. (ref. 19 in main text).

Analysis of ferroptosis by flow cytometry

Lipid peroxidation as a hallmark of ferroptosis was determined using the C11-BODIPY probe (D3861, Invitrogen, Carlsbad, CA, USA). Briefly, cells were incubated with 5 μM C11-BODIPY for 40 min. Then, both floating (dead) and attached (alive) cells were collected for each condition and run on a Gallios™ Cytometer (Beckman Coulter) at the Biology-Bellvitge Unit from Scientific and Technological Centers (CCiTUB), Universitat de Barcelona. The ferroptosis inducer RSL3 (S8155, Selleck Chemicals) was used at 2 μM as positive control

and incubated overnight. To determine viability, cells were stained with 4',6-diamidino-2-phenylindole (DAPI).

Gene signatures obtained from public data bases

ROS and TGF-β signaling gene signatures were obtained from Hallmark collection. Oxidative phosphorylation signature (In-house) was generated selecting genes that were in at least 2 out of the 3 oxidative phosphorylation signatures from MSigDB v2023.1 (Hallmark, WikiPathway, and Gene Ontology Biological Process collections).

Analysis of publicly available gene expression data

Single-cell RNA-sequencing data from Yang *et al.* cohort (ref. 30 in main text) was accessed through GEO accession number GSE171904 and the processed Seurat object was used to quantify gene expression signatures in non-parenchymal liver cell types with the AddModuleScore function. Comparisons between conditions were assessed using Mann-Whitney U-test and adjusted for multiple comparisons.

Gene expression from acute CCl_4 damage in mouse hepatocytes was assessed using Godoy *et al.* cohort (accessed through ArrayExpress E-MTAB-4444) (ref. 29 in main text). Raw data was downloaded and normalized using robust multi-array average (RMA) (1). Additionally, mouse liver gene expression data from chronic CCl_4-bulk sequencing (Hammad *et al.* cohort, ref. 31 in main text) was also downloaded and normalized using RMA through Gene Expression Omnibus (GEO) accession number GSE222576.

To analyze human gene expression, Fujiwara *et al.* cohort of liver biopsies from HCC-naïve MASLD patients (ref. 34 in main text) was accessed through Gene Expression Omnibus (GEO) accession number GSE193066. Relative log-expression normalized data was directly downloaded from GEO. Trepo *et al.* cohort of liver biopsies from HCC-naïve ASH and alcoholic cirrhosis patients (ref. 35 in main text) was accessed through GEO accession GSE103580 and raw data was downloaded and normalized using RMA.

For each cohort, a collection of gene signatures was obtained from MSigDB v2023 (2) and gene set variation analysis (GSVA) (3) was used to assess the relative activation of the signatures in the samples. Heatmaps showing relative activation for each gene signature were plotted using ComplexHeatmap package for R.

Correlation in human samples between two gene signatures was assessed using Pearson correlation. Kendall's τ was used to assess the association between the gene signatures and fibrosis stage in Fujiwara *et al.* cohort. All p-values were adjusted for multiple testing with Bonferroni test correction. All analyses were performed using R v4.0.4 (4).

Supplementary Table

Supplementary Table 1. Primers used for RT-qPCR

Species	Gene	Forward	Reverse
Homo sapiens	*CDKN1A*	CCAGCATGACAGATTTCTACCAC	GATGTAGAGCGGGCCTTTGA
Homo sapiens	*COL1A1*	CCCCTGGAAAGAATGGAGATG	TCCAAACCACTGAAACCTCTG
Homo sapiens	*FN1*	ACTGTACATGCTTCGGTCAG	AGTCTCTGAATCCTGGCATTG
Homo sapiens	*HAMP*	TTTTCCCACAACAGACGGGA	CTCCTTCGCCTCTGGAACAT
Homo sapiens	*HMOX1*	AGACACCCTAATGTGGCAGC	ATGGCCGTGTCAACAAGGAT
Homo sapiens	*RPL32*	AACGTCAAGGAGCTGGAAG	GGGTTGGTGACTCTGATGG
Homo sapiens	*TFR1*	TGAAGGTCTGACACGTCTGC	TGATGGTTCACTCACGGAGC
Mus musculus	*Hfe*	CTCAAAGCTGATGCCTCTGGA	CCAAGCCAAATGACAGCTTCC
Mus musculus	*Rpl32*	ACAATGTCAAGGAGCTGGAG	TTGGGATTGGTGACTCTGATG
Mus musculus	*Tf*	AGAACCGCTGGTTGGAACAT	GCGCAGCCTTGACTGAAAAA
Mus musculus	*Tfrc1*	GCACCACTCGCCCAAGTTAT	TGGAATCCCATTATGCACGGT
Mus musculus	*Tfrc2*	GGACTACTTGCAGAGTTCAGG	TTCCACGCGTCTGTAGATGG

Supplementary Figures

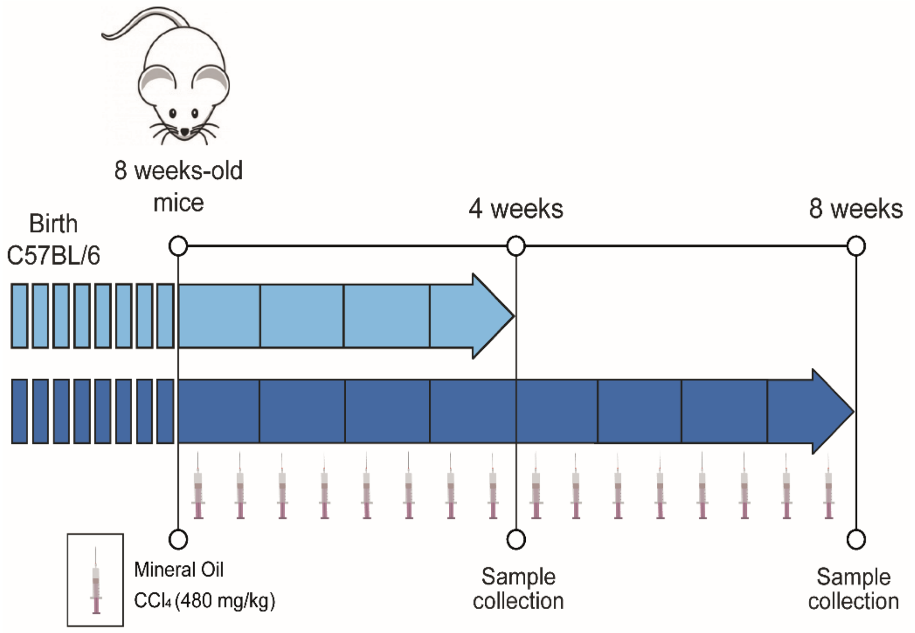

Supplementary Figure 1. Generation of a CCl_4-induced liver fibrosis murine model. Schematic representation of the experimental model used is shown. Eight-weeks old mice (C57BL/6 strain) were randomly divided into two groups (Vehicle and CCl_4). To induce liver fibrosis, we injected them intraperitoneally twice a week with a CCl_4 solution (480 mg/kg of body weight) or vehicle (mineral oil). After 4 or 8 weeks, mice were sacrificed, and liver tissues were collected for analysis.

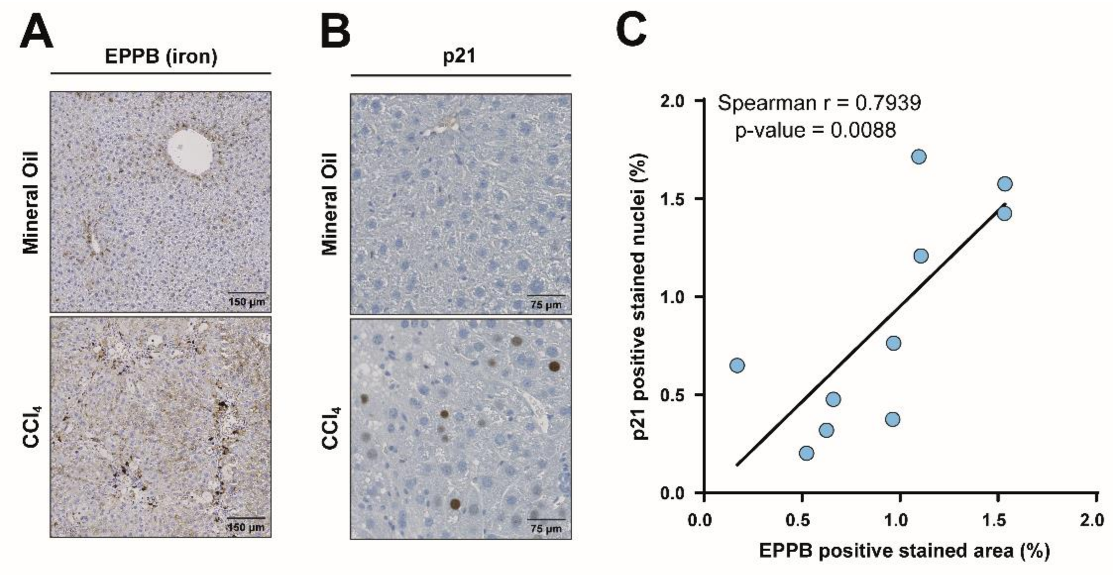

Supplementary Figure 2. Characterization of iron accumulation and senescence in a CCl_4-induced liver fibrosis murine model at 4 weeks. **A.** Enhanced Perl's Prussian Blue (EPPB) staining for iron detection. **B.** Immunohistochemistry for p21 as a hallmark of senescence. **C.** Spearman correlation analysis between the percentage of area stained by EPPB and the percentage of positive nuclei for p21, as analyzed by IHC. In EPPB staining, three representative regions from different liver lobes of each animal were selected and the percentage of positive stained area was quantified with ImageJ analysis software v1.44o. Then, the arithmetic mean of the percentage of stained area of the three regions was calculated for each animal. The percentage of p21-positive stained cells was quantified with QuPath software v0.4.4 in a whole liver lobe for each animal. Each dot represents a different animal (n=10). Statistical analysis was done using GraphPad Prism software (Spearman's correlation analysis).

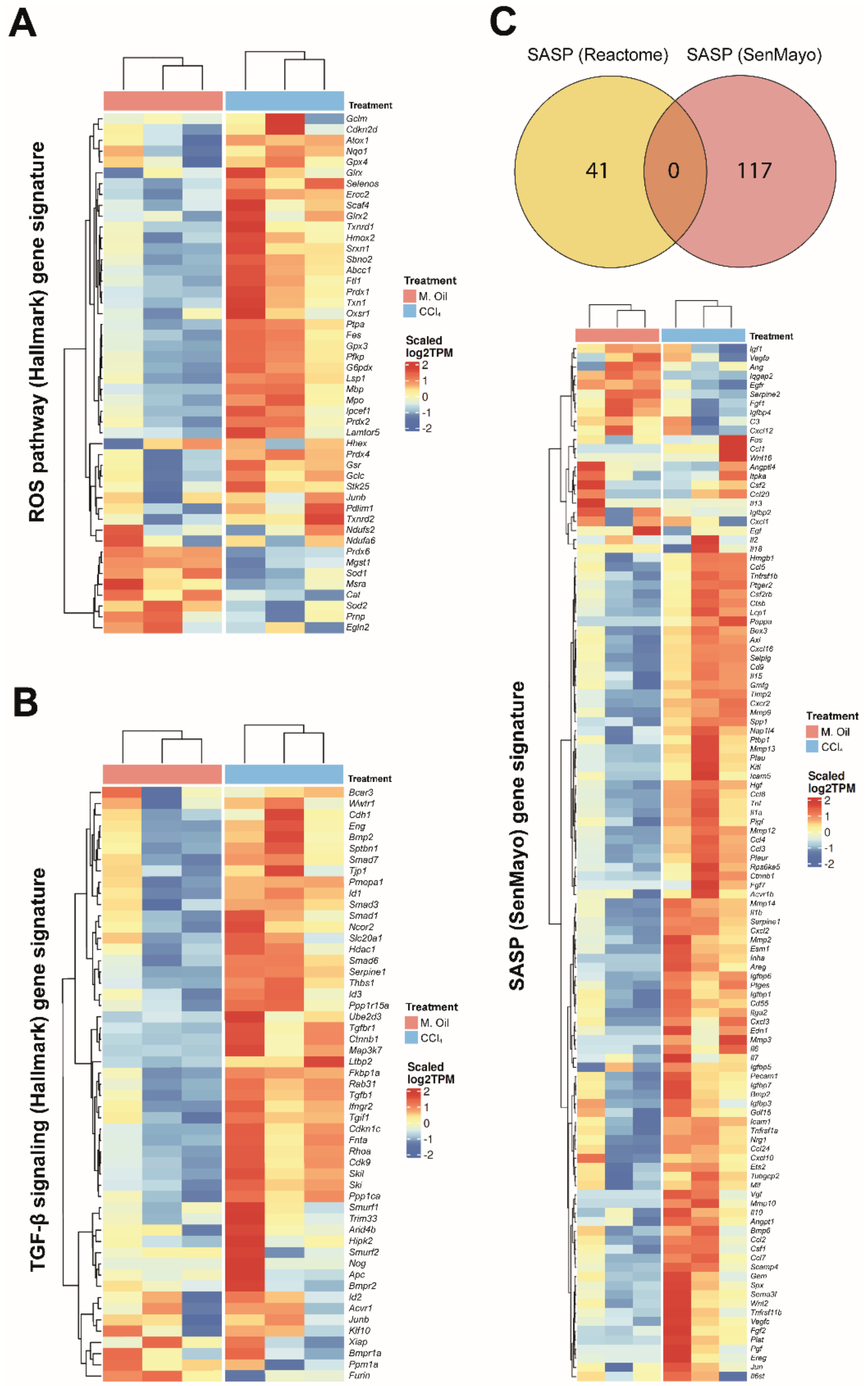

Supplementary Figure 3. Analysis of fibrosis- and SASP-related gene transcriptomes in hepatocytes from untreated or CCl_4-treated mice (complementary information to Fig. 2). RNA-seq analysis in hepatocytes from mice treated with CCl_4 or mineral oil (M. Oil) for 4 weeks (n=3 mice/group). Samples were tested for fibrotic hallmarks through ROS pathway (**A**) and TGF-β signaling (**B**) gene signatures from Hallmarks collection. **C.** Venn diagram showing the number of common mouse genes between the two SASP gene signatures (Reactome and SenMayo) tested in this study (**top**). Heatmap showing changes in gene expression from the SASP SenMayo gene signature in hepatocytes from CCl_4-treated mice compared to controls (**bottom**).

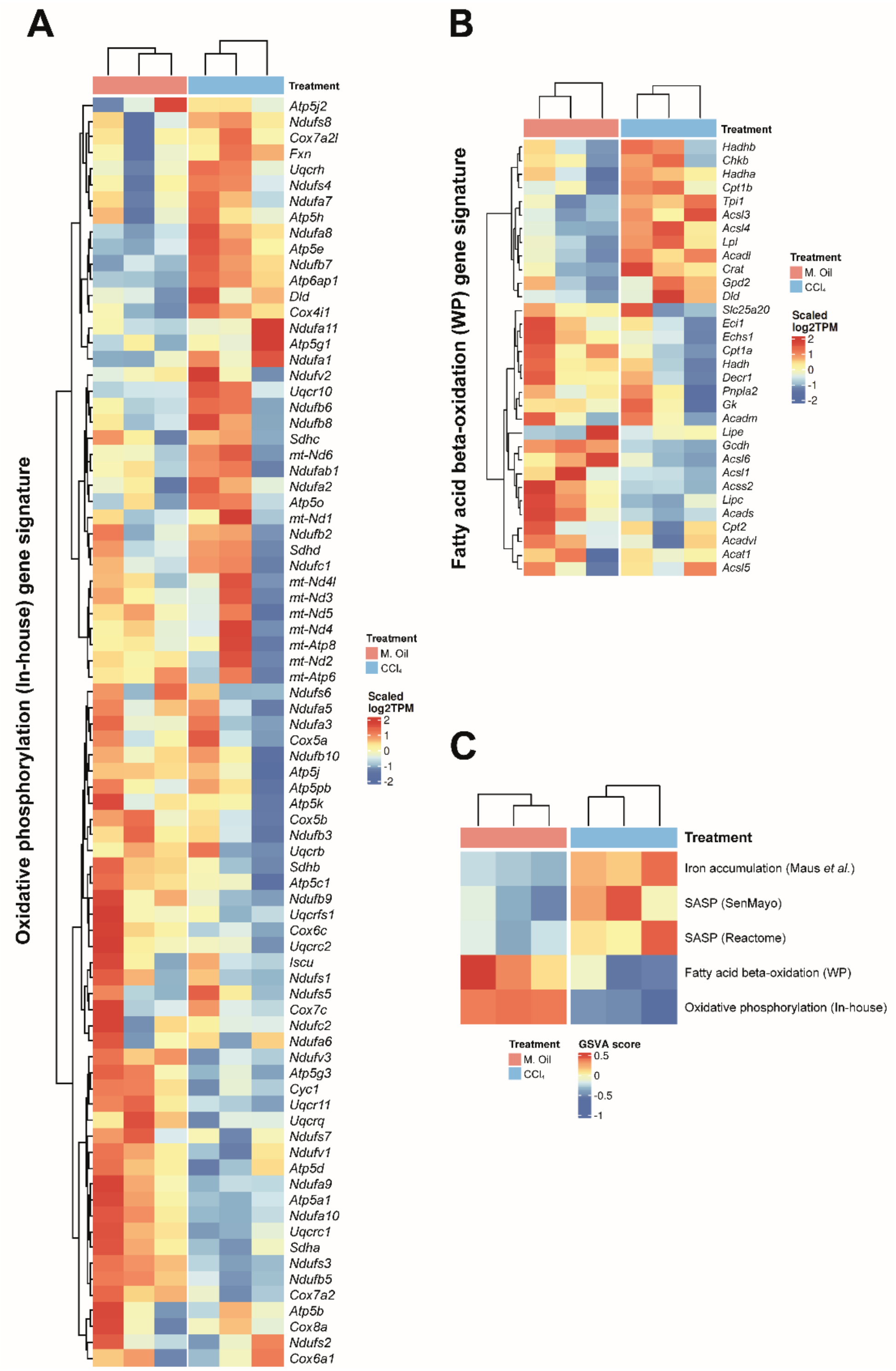

Supplementary Figure 4. Analysis of oxidative metabolism-related gene transcriptomes in hepatocytes from untreated or CCl_4-treated mice. RNA-seq analysis in hepatocytes from mice treated with CCl_4 or mineral oil (M. Oil) for 4 weeks (n=3 mice/group). **A.** Heatmap showing changes in gene expression for an oxidative phosphorylation gene signature (In-house) generated by selecting genes that were in at least 2 out of the 3 oxidative phosphorylation signatures from MSigDB v2023.1 (Hallmark, WikiPathways: WP, and Gene Ontology Biological Process: GOBP collections) **B.** Heatmap showing changes in gene expression for a fatty acid beta-oxidation gene signature (WP). **C.** Heatmap showing changes in relative activation of gene expression signatures analyzed with GSVA.

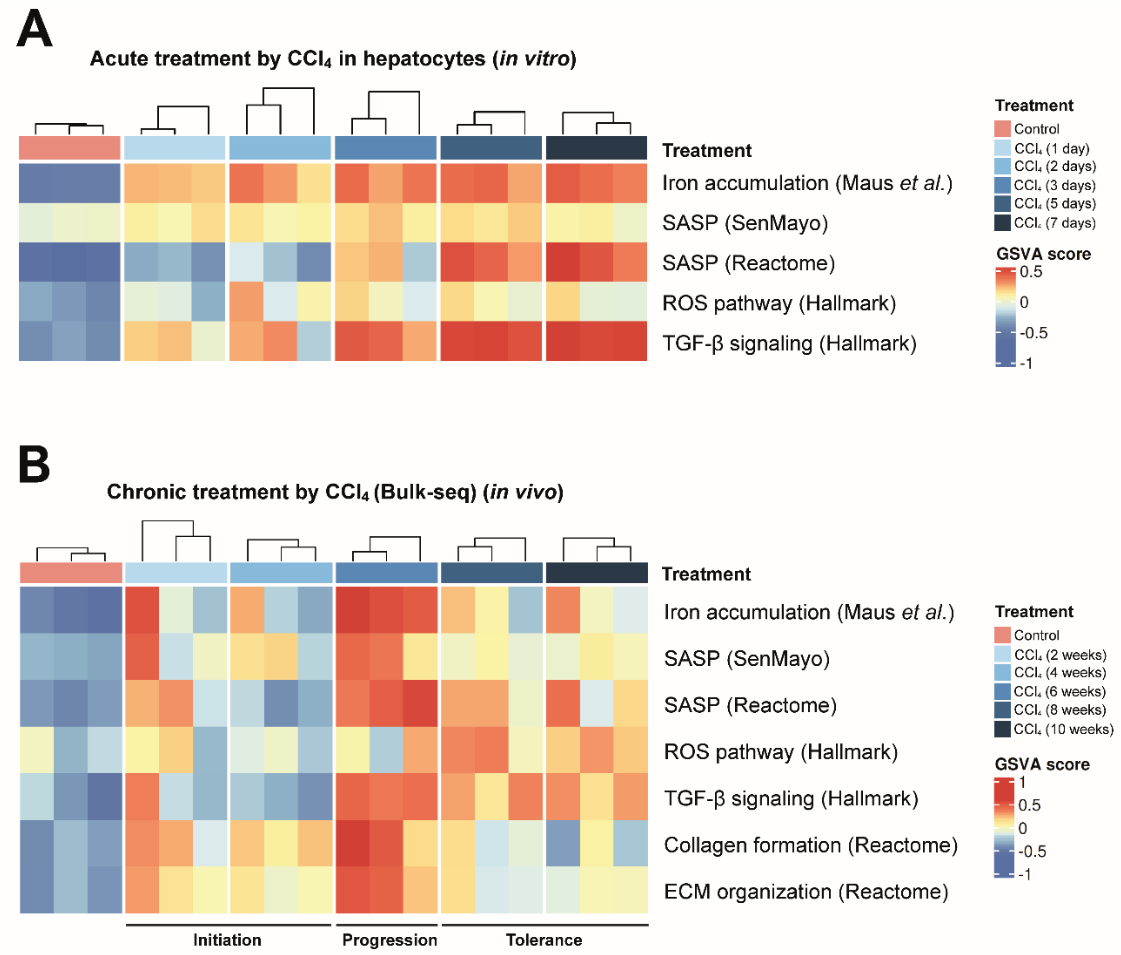

Supplementary Figure 5. Analysis of iron accumulation, SASP expression and fibrotic hallmarks induced by CCl4 in mouse. A. Heatmap showing changes in relative activation of gene expression signatures analyzed with GSVA in cultured mouse hepatocytes treated with CCl_4. **B.** Heatmap showing changes in relative activation of gene expression signatures analyzed with GSVA in bulk RNA isolated from mouse livers under chronic treatment with CCl_4 at different phases of fibrosis progression.

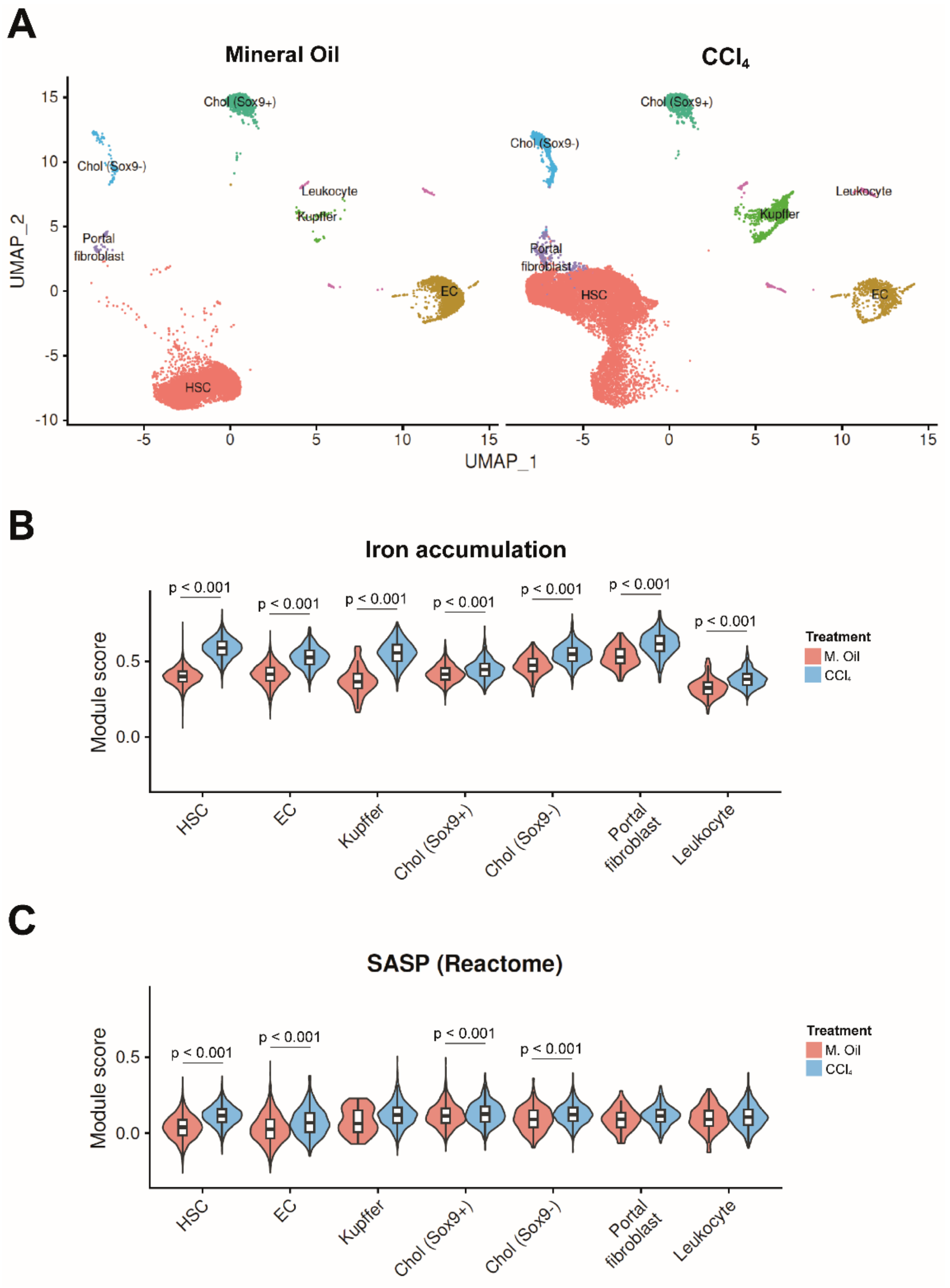

Supplementary Figure 6. Single-cell transcriptomic analysis of non-parenchymal liver cells from CCl_4-treated mice. A. UMAP visualization of non-parenchymal cell types in each treatment condition (n = 9 647 cells for Mineral Oil-treated and n = 16 774 cells for CCl_4-treated mice). **B-C**. Relative quantification of iron accumulation (**B**) and SASP (Reactome) (**C**) gene expression signatures in single-cell RNA-seq data from mineral oil- and CCl_4-treated mice. Statistical analysis was done using Mann-Whitney U-test and adjusted for multiple comparisons. HSC: hepatic stellate cells; EC: Endothelial Cells; Chol: Cholangiocytes.

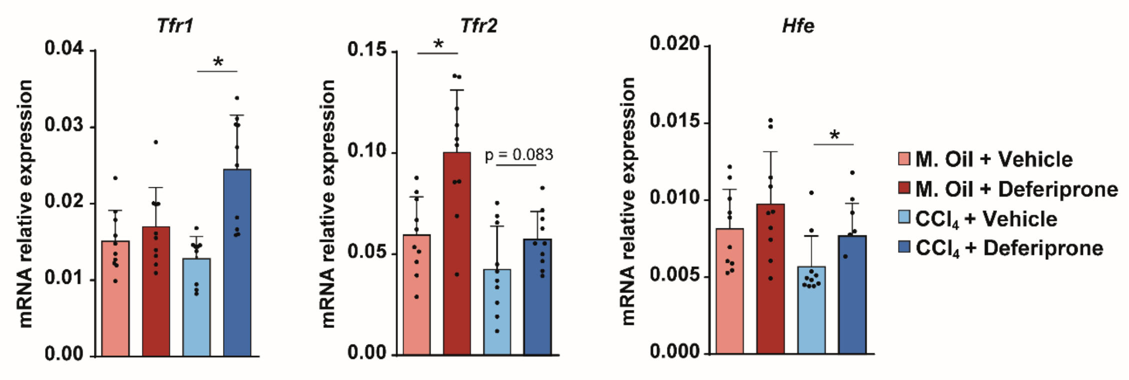

Supplementary Figure 7. Effects of deferiprone on gene expression related to iron metabolism in the model of CCl_4-induced liver fibrosis. RT-qPCR analysis of a panel of genes related to iron metabolism. Data are presented as mean (SD) (n=10 mice/group). Statistical analysis was done with two-tailed Mann-Whitney U-test to compare deferiprone treatment *versus* vehicle. *p<0.05.

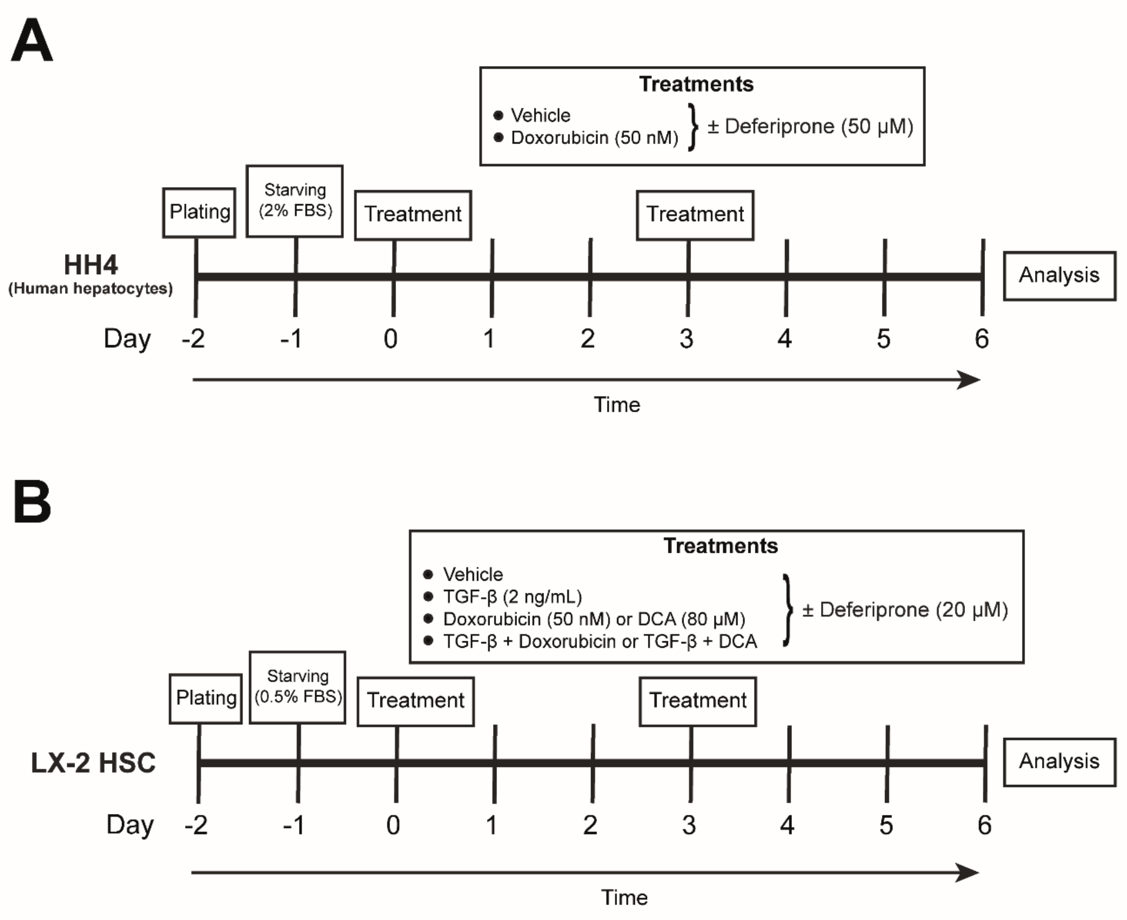

Supplementary Figure 8. Schematic representation of the experimental designs for *in vitro* cell culture experiments. **A.** The human hepatocyte cell line HH4 was cultured in MEM with 15% FBS and 24 h later starved with 2% of FBS. The next day (day 0), cells were treated with doxorubicin (50 nM) for 6 days to induce senescence and co-treated in the presence or absence of deferiprone (50 μM) for different analyses. **B.** LX-2 cell line was cultured in DMEM with 10% FBS and 24 h later starved with DMEM 0.5% FBS. The following day (day 0), cells were treated with vehicle, TGF-β (2 ng/mL), doxorubicin (50 μM) or deoxycholic acid (DCA, 80 μM) as senescent inducers, or the combination of TGF-β and the senescence inducer and maintained for 6 days in presence or absence of deferiprone (20 μM). In both A and B, media and treatments were refreshed at day 3.

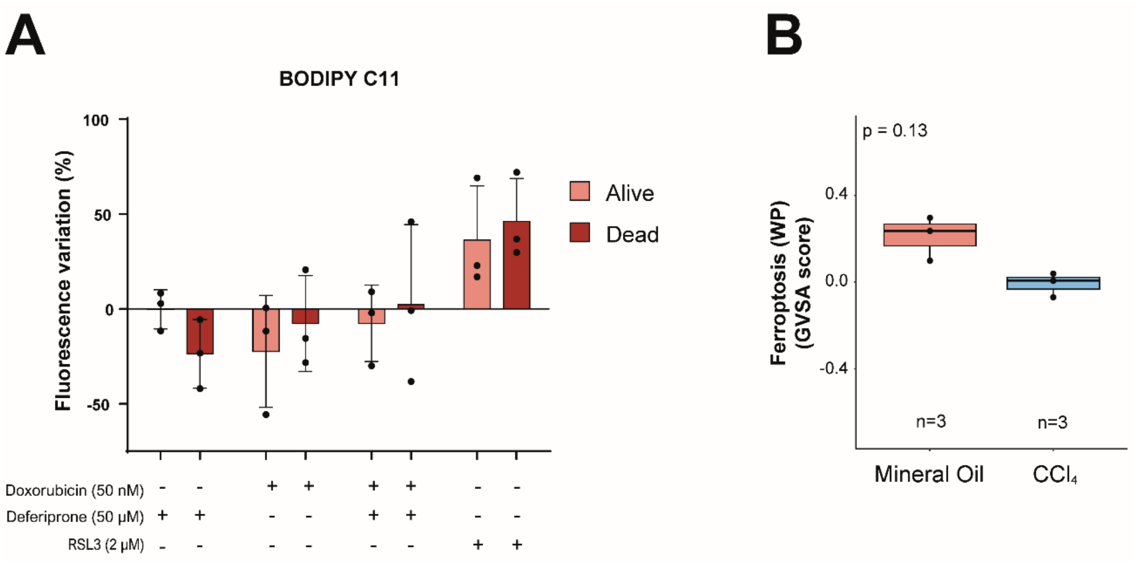

Supplementary Figure 9. Analysis of ferroptosis in human hepatocytes (*in vitro*) and murine hepatocytes isolated from the *in vivo* CCl_4 model. A. HH4 hepatocyte cell line was cultured as detailed in Supplementary Figure 8A and then incubated 40 min with BODIPY C11 (5 µM) probe as an indicator of lipid peroxidation linked to ferroptosis. Attached (alive) and floating (dead) cells were then collected, stained with DAPI to analyze cell viability, and run in a Gallios™ Cytometer. RSL3 (2 µM) treatment overnight was used as positive control. Three independent experiments were done. Results are shown as mean (SD) expressed as percentage of fluorescence variation *versus* control cells (untreated). **B.** Boxplot of relative enrichment for the ferroptosis gene signature in hepatocytes isolated from mice treated with CCl_4 or mineral oil for 4 weeks, analyzed by RNA-seq (n=3 mice/group). Statistical analysis was done with two-tailed Mann-Whitney U-test.

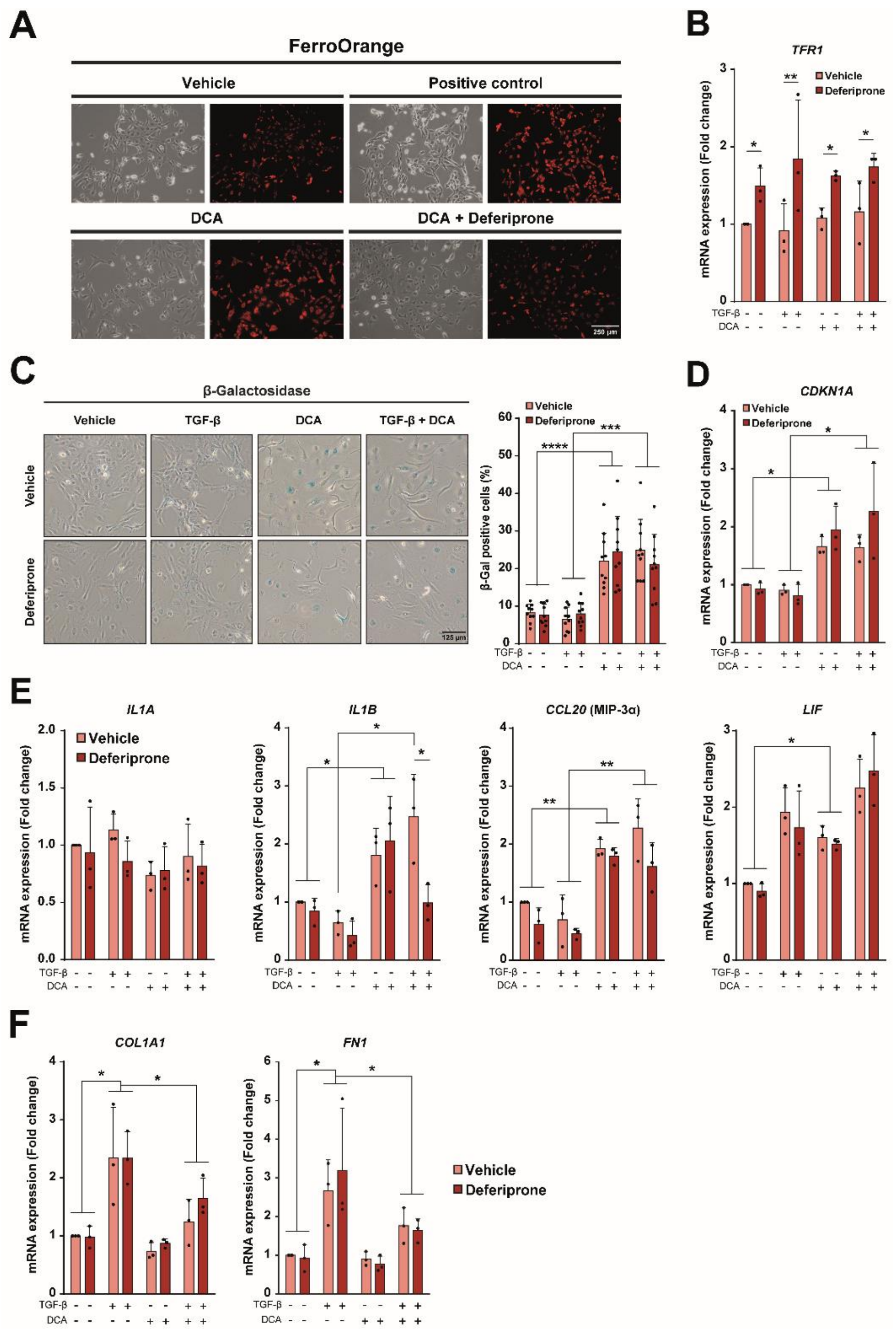

Supplementary Figure 10. Effects of deferiprone on iron accumulation, senescence and activation markers in human HSC. The human HSC cell line LX-2 was cultured as detailed in Supplementary Figure 8B with DCA as a senescent inducer. **A.** FerroOrange dye was used to analyze iron accumulation. Treatment for 24 h with iron (660 µM) was used as positive control. Images were taken with a fluorescence microscope and representative ones are shown. **B.** *TFR1* mRNA levels were analyzed by RT-qPCR. **C.** β-Galactosidase staining assay was performed to analyze senescence. Ten images were taken from random fields by phase contrast microscopy (left) and percentage of positive cells were quantified (right). **D-F.** Analysis of mRNA levels by RT-qPCR of *CDKN1A*, a panel of SASP related genes (*IL1A*, *IL1B*, *CCL20* and *LIF*) and HSC activation related genes (*COL1A1*, *FN1*). All analyses in panels A-F were done at day 6 post-treatment. Statistical analysis was done with one-way ANOVA with Sidak's correction (n=3 independent experiments). In A and C, a representative experiment is shown *$p<0.05$; **$p<0.01$; ***$p<0.001$; ****$p<0.0001$.

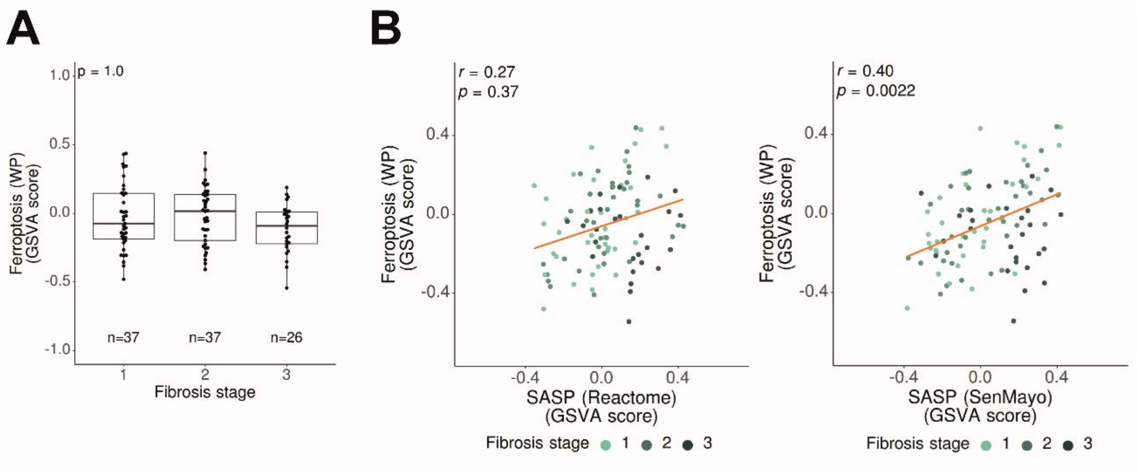

Supplementary Figure 11. Transcriptomic analysis of ferroptosis in a human cohort of liver fibrosis. A. Boxplot of relative enrichment (GSVA score) for the ferroptosis (WikiPathways: WP) gene signature across fibrosis stages in the Fujiwara *et al.* cohort of liver fibrosis patients (ref. 34 in main text). **B.** Pearson correlation analyses between the relative enrichment of both SASP signatures (Reactome and SenMayo) and the ferroptosis signature. Each dot is a sample (color indicates the fibrosis stage). Kendall's τ was used to assess the association between the gene signatures and fibrosis stage. P-values were adjusted using Bonferroni test correction.

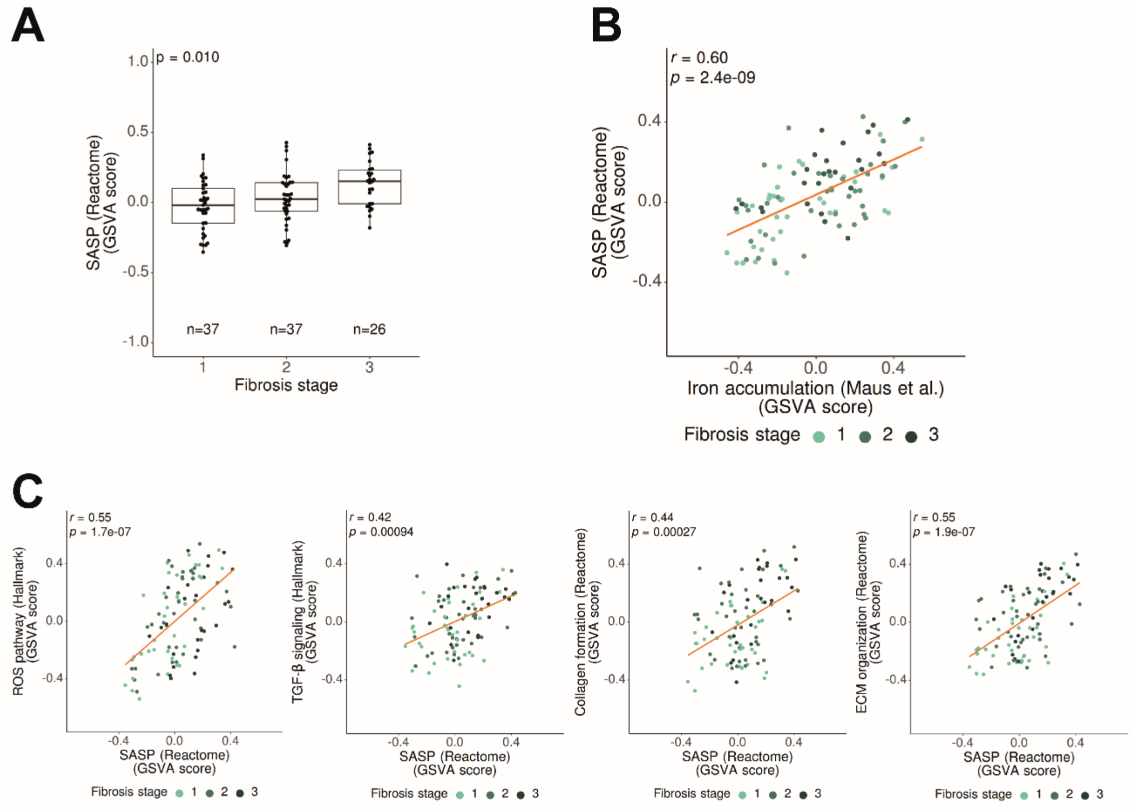

Supplementary Figure 12. Analysis of iron accumulation and SASP expression across liver fibrosis stages in human patients. A. Boxplot of SASP (Reactome) gene signature relative enrichment (GSVA score) across fibrosis stages in the Fujiwara *et al.* cohort of liver fibrosis patients (ref. 34 in main text). **B.** Pearson correlation analysis of the relative enrichment of iron accumulation gene signature with SASP (Reactome) gene signature. **C.** Pearson correlation analyses of the relative enrichment of SASP (Reactome) gene signature with ROS pathway (Hallmark), TGF-β signaling (Hallmark), collagen formation (Reactome) and ECM organization (Reactome) gene signatures. Each dot is a sample (color indicates the fibrosis stage). Kendall's τ was used to assess the association between the gene signatures and fibrosis stage. All analyses were adjusted for multiple testing with Bonferroni test correction.

References for Supplementary Information

1. Irizarry RA, Hobbs B, Collin F, Beazer-Barclay YD, Antonellis KJ, Scherf U, et al. Exploration, normalization, and summaries of high density oligonucleotide array probe level data. Biostatistics. 2003;4(2):249-64.
2. Liberzon A, Birger C, Thorvaldsdottir H, Ghandi M, Mesirov JP, Tamayo P. The Molecular Signatures Database (MSigDB) hallmark gene set collection. Cell Syst. 2015;1(6):417-25.
3. Hanzelmann S, Castelo R, Guinney J. GSVA: gene set variation analysis for microarray and RNA-seq data. BMC Bioinformatics. 2013;14:7.
4. R Core Team. R: A language and environment for statistical computing: R Foundation for Statistical Computing; 2021 [Available from: https://www.R-project.org/].
