# Supplementary material for: Iron chelation as a new therapeutic approach to prevent senescence and liver fibrosis progression
Source: Cell Death Dis. 2024 Sep 17;15(9):680. doi: 10.1038/s41419-024-07063-0 (PMC11408630; doi:10.1038/s41419-024-07063-0)

Supplementary uncropped blots

Iron chelation as a new therapeutic approach to prevent senescence and liver fibrosis progression

Josep Amengual[1,2], Ania Alay[3,4], Javier Vaquero[1,2,5], Ester Gonzalez-Sanchez[1,2,5,6], Esther Bertran[1,2], Aránzazu Sánchez[7,8] , Blanca Herrera[7,8], Kathleen Meyer[9,10], Mate Maus[9,11], Manuel Serrano[9,10,12], María Luz Martínez-Chantar[2,13], Isabel Fabregat[1,2#]

[1]TGF-β and Cancer Group. Oncobell Program, Bellvitge Biomedical Research Institute (IDIBELL), L'Hospitalet de Llobregat, Barcelona, Spain.

[2]Centro de Investigación Biomédica en Red de Enfermedades Hepáticas y Digestivas (CIBERehd), Madrid, Spain.

[3]Unit of Bioinformatics for Precision Oncology, Catalan Institute of Oncology (ICO), L'Hospitalet de Llobregat, Barcelona, Spain.

[4]Preclinical and Experimental Research in Thoracic Tumors (PReTT), Oncobell Program, IDIBELL, L'Hospitalet de Llobregat, Spain.

[5]HepatoBiliary Tumours Lab, Centro de Investigación del Cáncer and Instituto de Biología Molecular y Celular del Cáncer, CSIC-Universidad de Salamanca, Salamanca 37007, Spain.

[6]Department of Physiology and Pharmacology, University of Salamanca, 37007, Salamanca, Spain

[7]Department of Biochemistry and Molecular Biology, Faculty of Pharmacy, Complutense University of Madrid, Madrid, Spain.

[8]Health Research Institute of the “Hospital Clínico San Carlos” (IdISSC), Madrid, Spain.

[9]Institute for Research in Biomedicine (IRB Barcelona), The Barcelona Institute of Science and Technology (BIST), Barcelona, Spain.

[10]Altos Labs, Cambridge Institute of Science, Cambridge, United Kingdom.

[11]Vall d'Hebron Institute of Oncology, Barcelona, Spain.

[12]Catalan Institution for Research and Advanced Studies (ICREA), Barcelona, Spain.

[13]Liver Disease and Liver Metabolism Laboratory, CIC bioGUNE-BRTA (Basque Research & Technology Alliance), Derio, Bizkaia, Spain.

#Corresponding author: Isabel Fabregat. Email: ifabregat@idibell.cat

Original uncropped images (Figure 4C)

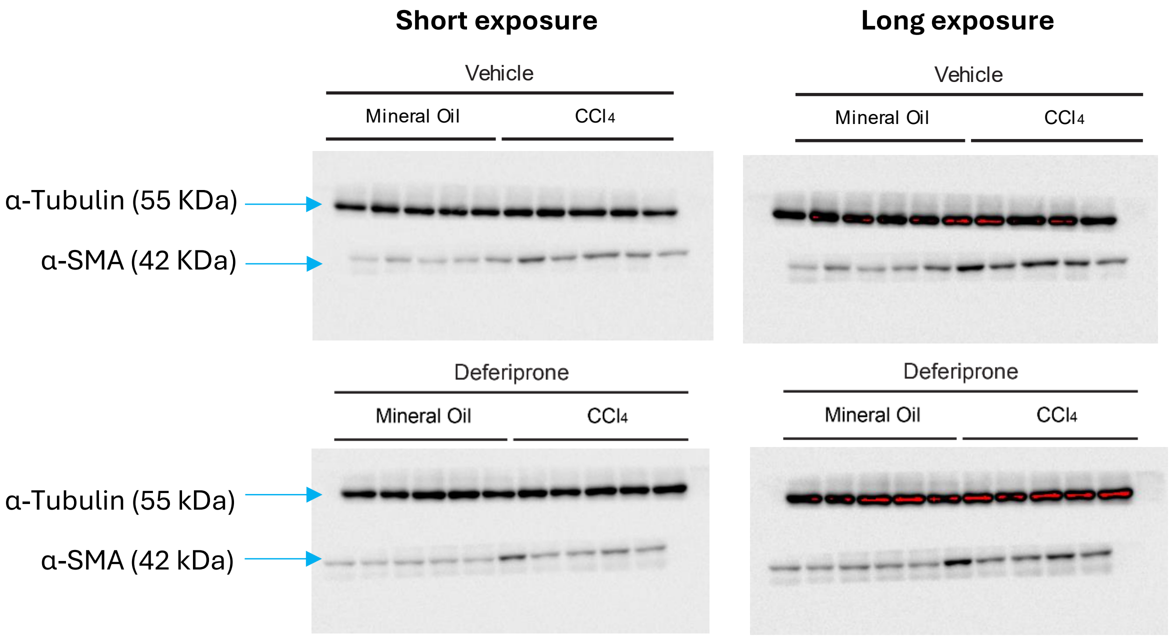

Supplement: Supplementary file 2 — Supplementary uncropped western blots [file 41419_2024_7063_MOESM2_ESM.pdf]
