# Supplementary material for: Iron chelation as a new therapeutic approach to prevent senescence and liver fibrosis progression
Source: Cell Death Dis. 2024 Sep 17;15(9):680. doi: 10.1038/s41419-024-07063-0 (PMC11408630; doi:10.1038/s41419-024-07063-0)

Applied Biosystems

GeneMapper v3.7

M389

Sample File	Sample Name	Panel	SQI	OS	SQ
frag_007_D07.fsa	02.I.Fabregat_LX2	GenePrint_10_v1.0			

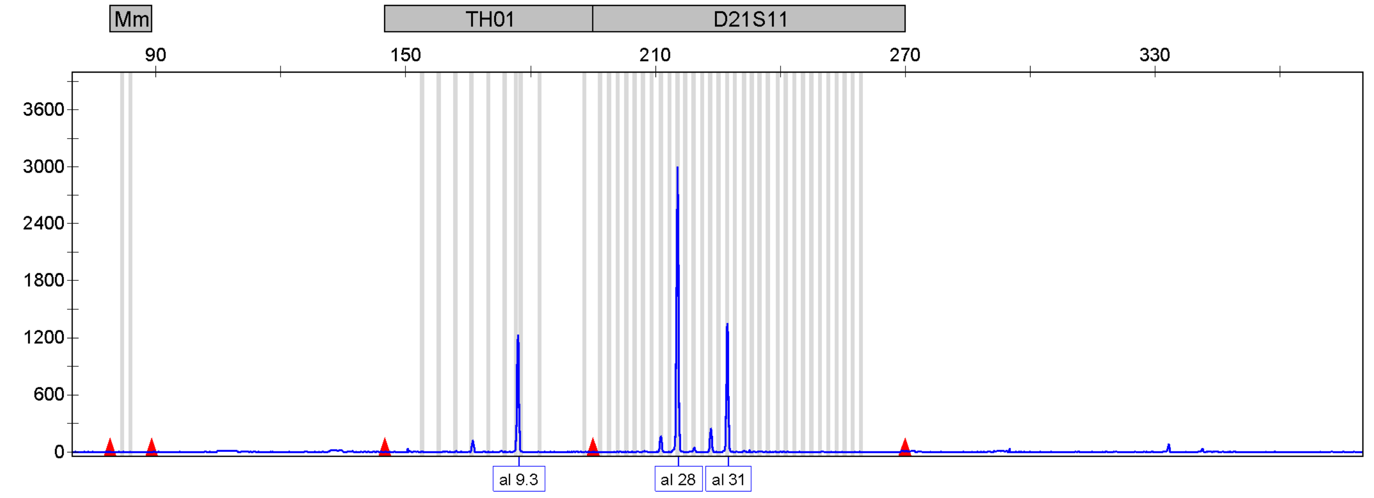

frag_007_D07.fsa	02.I.Fabregat_LX2	GenePrint_10_v1.0			

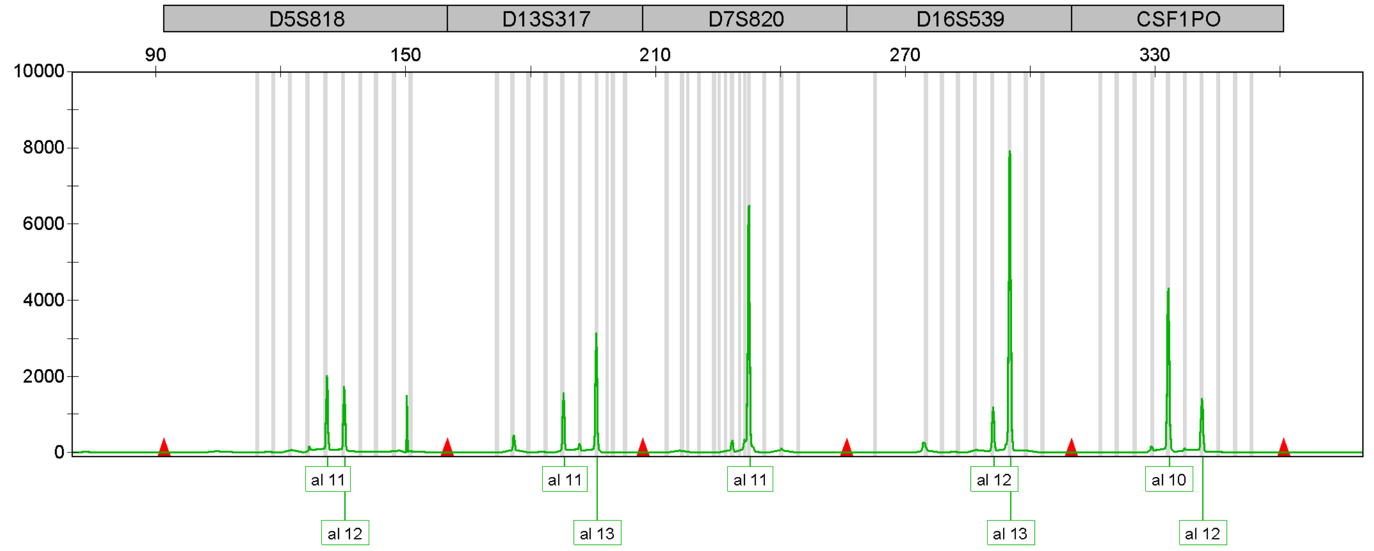

frag_007_D07.fsa	02.I.Fabregat_LX2	GenePrint_10_v1.0			

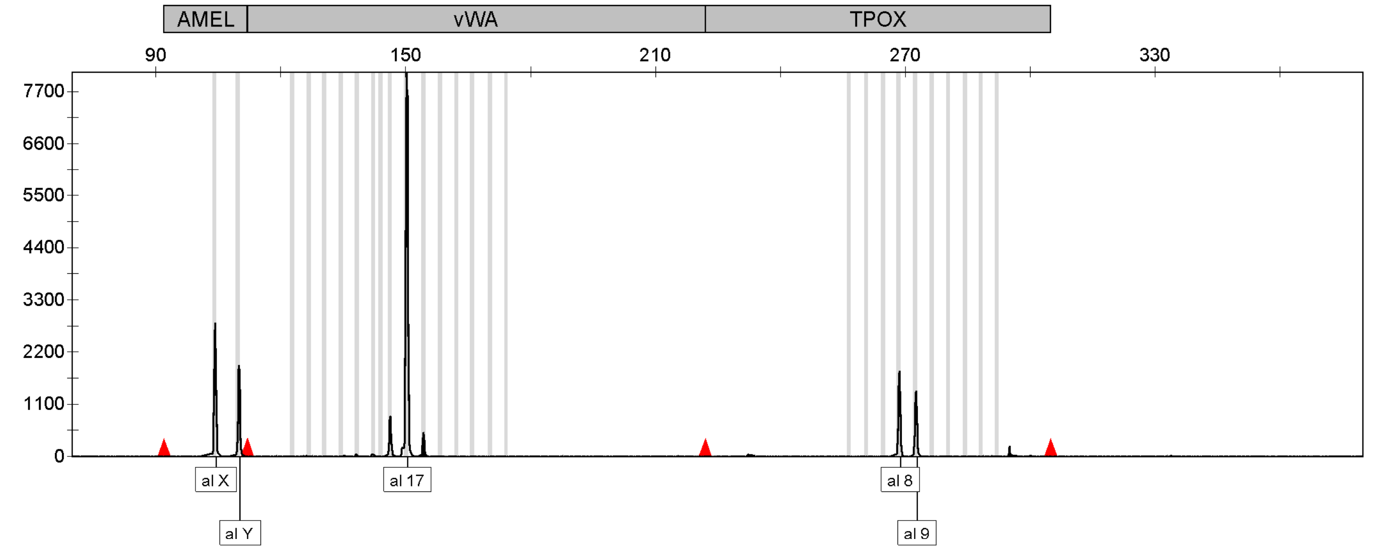

Supplement: Supplementary file 3 — Report for authentication LX-2 cells [file 41419_2024_7063_MOESM3_ESM.pdf]
